# Deep learning models for pancreatic cancer detection on CT: a meta-analysis

**DOI:** 10.3389/fmed.2026.1826557

**Published:** 2026-09-14

**Authors:** Yunxia Ding, Han Qin, Zhen Qu, Jiangyi Ju, Lihua Peng

**Affiliations:** Bishan Hospital of Chongqing Medical University, Chongqing, China

**Keywords:** CT scans, deep learning, diagnostic accuracy, early detection, pancreatic ductal adenocarcinoma, systematic review and meta-analysis

## Abstract

This systematic review and meta-analysis evaluates the diagnostic accuracy of deep learning models for detecting pancreatic ductal adenocarcinoma using computed tomography scans. A systematic literature search of PubMed, Web of Science, and the Cochrane Library was conducted for studies published up to September 11, 2025. Study screening and data extraction were performed independently by two reviewers. Studies utilizing deep learning models for pancreatic ductal adenocarcinoma detection on computed tomography, with histopathological confirmation as the reference standard, were included. Studies employing solely traditional machine learning models were excluded. Primary outcomes were sensitivity, specificity, diagnostic odds ratio, and area under the curve. Quality assessment was performed using standardized tools for risk of bias and methodological soundness. A subgroup analysis focused on scans acquired 3 to 36 months prior to clinical diagnosis. Fifteen studies were included. The pooled results demonstrated a sensitivity of 0.92 (95% confidence interval: 0.88–0.94), specificity of 0.96 (95% CI: 0.92–0.98), diagnostic odds ratio of 285.00 (95% CI, 97.00–839.00), and area under the curve of 0.97 (95% CI, 0.95–0.98). In the preclinical diagnosis subgroup, the pooled sensitivity was 0.73 and specificity was 0.92. This meta-analysis validates the high diagnostic accuracy of deep learning models for detecting pancreatic cancer on CT scans in retrospective datasets. However, all included studies were retrospective, external validation was inconsistent, and no prospective or real-world deployment evidence exists to date. Therefore, while these findings suggest the potential of DL as a decision-support tool, they should be interpreted with caution, and prospective multicenter validation studies are urgently needed before clinical integration can be recommended. However, the pooled estimates should be interpreted cautiously because only one representative model per study was included.

## 1 Introduction

Pancreatic ductal adenocarcinoma (PDAC), commonly known as pancreatic cancer, is a devastating disease with a grim prognosis. Between 2015 and 2019, the incidence rates of pancreatic cancer increased by 1 to 2% annually (1). Despite being the 12th most common cancer in the United States, it is projected to become the second leading cause of cancer-related death by 2030 owing to its aggressive nature and late-stage diagnosis (2). The 5-year relative survival rate for pancreatic cancer stands at a dismal 13%, among the lowest of all cancers, highlighting the urgency for improved early detection and treatment strategies. PDAC also significantly adds to the economic burden on individuals and society, with an estimated cost in the United States between $421 billion and $451 billion in 2018 (3, 4).

Radical surgery remains the cornerstone treatment for patients with localized PDAC, offering the only potential for long-term survival (5). However, the effectiveness of surgical intervention is heavily reliant on the stage at which the cancer is detected. Unfortunately, early diagnosis of pancreatic cancer is exceedingly difficult because of several critical factors. First, pancreatic cancer often presents with nonspecific symptoms, such as abdominal pain, weight loss, and jaundice, which are easily mistaken for other less severe conditions. These symptoms typically do not appear until the disease has progressed to an advanced stage, making early detection a significant challenge. Additionally, the anatomical location of the pancreas deep within the abdomen further complicates the identification of early-stage tumors through routine physical examinations or imaging studies. Moreover, the variability in diagnostic accuracy among radiologists adds another layer of complexity to the early detection of pancreatic cancer. Imaging modalities such as computed tomography (CT) and magnetic resonance imaging (MRI) are commonly used for diagnosis, but the interpretation of these images can vary significantly among different radiologists. This inconsistency can lead to missed diagnoses or delayed detection, ultimately affecting patient prognosis and treatment options (6–8). Given these challenges, the majority of patients are diagnosed at an advanced stage when the cancer has already metastasized, rendering radical surgery ineffective. As a result, the opportunity for curative treatment is often lost, contributing to the high mortality rate associated with pancreatic cancer. These findings underscore the urgent need to develop a diagnostic model for PDAC that offers increased sensitivity and specificity. Such a model could identify early-stage disease, allowing timely intervention and improving the chances of successful treatment and long-term survival for patients with PDAC.

Deep learning algorithms, such as convolutional neural networks (CNNs) and transformers, have demonstrated remarkable capabilities in the field of medical image analysis with high precision (9). These algorithms are trained on large volumes of annotated medical imaging data, enabling them to learn the characteristic patterns of different tissues, organs, and lesions within images. During the training process, deep learning models automatically extract key features from images, such as shape, texture, density, and contrast, and match these features against known pathological states or normal appearances (10, 11). Once the model is trained, it can precisely delineate the boundaries of structures of interest, such as tumors, blood vessels, and cells, thereby enabling the analysis of new imaging data to rapidly identify abnormal areas (such as tumors, inflammation, or other lesions) and provide diagnostic recommendations or segmentation results (12). In cancer detection, deep learning models have shown high sensitivity and specificity in identifying tumors, significantly improving diagnostic accuracy (13). This capability is crucial for early disease detection, as it allows for timely intervention, thereby increasing the chances of successful treatment and long-term survival. In particular, in the diagnosis of pancreatic cancer, the application of deep learning models on CT images has been proven feasible (14). Pancreatic cancer often presents with nonspecific symptoms and progresses rapidly, leading to late-stage diagnosis, which makes treatment challenging and the prognosis poor (3). Through the analysis of CT images, deep learning models can quickly identify subtle abnormalities and early signs of tumors in the pancreas, helping doctors detect pancreatic cancer earlier and providing more timely treatment plans for patients. Moreover, the efficiency and accuracy of deep learning models can significantly reduce the workload of medical personnel, optimize clinical decision-making processes, lower patient costs, and drive the development of intelligent and efficient medical services (14–16).

Although previous systematic reviews and meta-analyses have explored the value of artificial intelligence in the diagnosis of PDAC, such as the study by Harandi et al. (17), which included various AI algorithms (e.g., random forest, support vector machines), their research scope was broad and not specifically focused on deep learning models, nor did they sufficiently incorporate large-scale, prospective studies published from 2023–2024. Another meta-analysis by Costa et al. (18) focused on the performance of machine learning models in contrast-enhanced CT but included only 9 studies with a limited sample size. It does not specifically evaluate deep learning architectures (e.g., CNNs or transformers) or systematically analyze their potential in early PDAC detection.

Therefore, through this systematic review and meta-analysis, our aim is to specifically evaluate the accuracy of deep learning models in diagnosing pancreatic ductal adenocarcinoma (PDAC) on CT, incorporating the latest studies up to September 2025, to address the gaps in the literature regarding model type, timeliness, and diagnostic performance assessment.

Several recent meta-analyses have reported high pooled sensitivity and specificity for AI-based PDAC detection. Harandi et al. (17) included 26 studies that mixed conventional machine learning (e.g., random forest, support vector machines) with deep learning models, reporting a pooled sensitivity of 93% and specificity of 95% on internal validation. Costa et al. (18) included nine studies (mostly deep learning) and reported a sensitivity of 87.9% and specificity of 92.2%. However, these meta-analyses did not isolate pure deep learning architectures, did not compare the performance of different DL architectures (pure CNN vs. hybrid vs. task-enhanced), and did not quantitatively synthesize prediagnostic (early) detection performance due to their search cutoffs or exclusion criteria. Furthermore, none applied the recently developed METRICS tool to assess methodological quality at the item level. Therefore, our study fills these gaps by: (i) focusing exclusively on deep learning; (ii) providing the first head-to-head comparison of DL architectures; (iii) reporting the first pooled estimates of prediagnostic performance; and (iv) using four complementary quality assessment tools, including METRICS, to identify actionable methodological deficiencies.

## 2 Materials and methods

This meta-analysis followed the Preferred Reporting Items for Systematic Reviews and Meta-Analysis (PRISMA) guidelines for conducting and reporting a study (19). This study was prospectively registered in PROSPERO (CRD42025625942).

### 2.1 Literature search strategy

Two observers independently searched the PubMed, Cochrane Library and Web of Science databases to identify studies published up until September 11, 2025. To conduct a comprehensive literature search and include an engineering and technology database, IEEE was also searched until the same date. The keywords we searched included “pancreatic cancer,” “pancreatic ductal cell carcinoma,” “artificial intelligence,” “deep learning,” “convolutional neural network,” “computed tomography,” etc. Two reviewers (Y.D. and H.Q.) independently performed a comprehensive literature search in PubMed, Web of Science, the Cochrane Library, and IEEE Xplore from database inception to September 11, 2025. Controlled vocabulary (e.g., MeSH terms) and free-text keywords were combined using Boolean operators (“AND” and “OR”). Specifically, terms related to pancreatic cancer were combined with terms describing artificial intelligence/deep learning and computed tomography (CT). The detailed search strategies for each database are provided in Supplementary Table 1. No language restriction was adopted.

### 2.2 Study selection

After duplicate records were removed, two reviewers independently screened the titles and abstracts of all retrieved records for potential eligibility. Full texts of potentially eligible studies were subsequently assessed independently according to the predefined inclusion and exclusion criteria. Any disagreements between the reviewers were resolved through discussion until consensus was reached. If necessary, a third reviewer was consulted to resolve unresolved disagreements.

According to the PICOS framework, studies meeting the following criteria were included: (1) study participants were patients diagnosed with PDAC; (2) study participants had CT imaging data acquired prior to the clinical diagnosis of pancreatic cancer; (3) studies used histopathological results as the reference standard; and (4) studies provided sufficient data to construct a 2 × 2 contingency table for analysis.

Studies were excluded on the basis of the following criteria: (1) reviews, case reports, letters, comments, errata, meta-analyses, and studies published solely as conference abstracts; (2) studies lacking internal or external validation; and (3) studies that employed only traditional machine learning models (e.g., random forest or support vector machines) without involving deep learning architectures.

### 2.3 Data extraction

Terminology clarification. In this study, “deep learning” (DL) refers specifically to multi-layer neural network architectures (e.g., convolutional neural networks, U-Nets, and Transformer-based models), whereas “artificial intelligence” (AI) is used only in a general sense to denote the broader field. The term “model” denotes an individual algorithmic implementation evaluated within a study. “Detection” and “diagnosis” are used to describe the identification of pancreatic ductal adenocarcinoma (PDAC) on CT images at the time of clinical presentation, reflecting the primary aim of this meta-analysis. In contrast, “prediagnostic detection” refers to the identification of PDAC on CT scans acquired prior to clinical diagnosis (e.g., months to years before symptom onset). These definitions are applied consistently throughout the manuscript to avoid ambiguity.

The following information was extracted independently by the same investigators: (1) general information on the study (lead author, year of publication, country, study type, study interval, sample size and characteristics of patients), (2) technical parameters of CT, (3) reference standard, (4) deep learning model, and (5) 2 × 2 tables (with true positive, true negative, false positive, and false negative values).

When different deep learning models were tested within the same paper, the proposed model with the highest area under the curve (AUC) was selected for the main analysis. If no AUC was provided, the model with the highest diagnostic accuracy was chosen. This hierarchical rule was pre-specified to avoid post-hoc selection bias. This approach follows the reductionist strategy for handling effect size multiplicity, wherein a single representative effect size is selected per study to preserve the independence assumption across effect sizes (20).

Our study included data from external validation sets and test sets, defined as independent case collections not used to determine any preprocessing methods or model optimization. If the original study used cross-validation, the entire cohort was included in the analysis.

The data were cross-checked, and any discrepancies were discussed.

### 2.4 Technical and mathematical foundations of the included deep learning models

To facilitate understanding of the architectural differences among the included studies, we summarise the core mathematical principles of the major deep learning models identified in our meta-analysis, with explicit references to the studies that employed each architecture.

#### 2.4.1 Convolutional neural networks (CNNs)

The fundamental operation of a CNN is discrete convolution. For a 2D image II and a kernel KK, the convolution at position (i,j)(i,j) is defined as:

$$\left(I*K)(i,j)=\sum_{m=0}^{M-1}\sum_{n=0}^{N-1}I(i+m,j+n)K(m,n\right)$$

This operation allows the network to learn spatial hierarchies of local features (e.g., edges, textures, intensity changes). 3D CNNs extend the convolution kernel to three dimensions to preserve volumetric context. In our review, 2D CNNs were used by Liu et al. (21) (VGG-style), Ma et al. (22) (three-layer CNN), Nadeem et al. (23) (11-layer AlexNet), Thanya et al. (24) (ResNet-50 for feature extraction), and Si et al. (25) (ResNet18/34 for localisation and diagnosis). 3D CNNs were employed by Korfiatis et al. (26), Qu et al. (27), Park et al. (28), and Ramaekers et al. (29) to capture the volumetric characteristics of the pancreas and surrounding structures.

#### 2.4.2 Residual networks (ResNet)

ResNet introduces an identity skip connection, formulated as:

$$y=F(x,\{W_{i}\})+x$$

Where x is the input, F represents the residual mapping to be learned, and y is the output. This design allows gradients to flow directly through the network, mitigating the vanishing gradient problem and enabling the construction of very deep models (30). In our review, ResNet architectures were used by Si et al. (25) (ResNet18/34), Thanya et al. (24) (ResNet-50), and Korfiatis et al. (26) (a modified ResNet within their 3D-CNN).

#### 2.4.3 U-net and nnU-net

U-Net consists of a symmetric encoder-decoder with skip connections (31). The encoder downsamples the input to capture high-level semantic features, while the decoder upsamples to recover spatial resolution. Skip connections transmit fine-grained positional information from the encoder to the decoder, enabling precise pixel-wise localisation. nnU-Net automatically configures its architecture, preprocessing, and training hyperparameters based on the characteristics of the dataset (32). In our review, 3D nnU-Net was used by Park et al. (28) and Mandal et al. (33); a multi-stage 3D U-Net was used by Ramaekers et al. (29) to segment the pancreas, pancreatic duct, and common bile duct; and U-Net32 was used by Si et al. (25) for pancreas segmentation.

#### 2.4.4 Vision transformers (ViTs) and hybrid CNN-transformer models

The core of a ViT is the self-attention mechanism (34):

$$\text{Attention}(Q,,K,,V)=\text{softmax}(\frac{{\mathrm{QK}}^{T}}{\sqrt{d_{k}}})V$$

where Q, K, and V are the query, key, and value matrices derived from the input, and dk is the dimension of the keys. Self-attention allows the model to capture long-range dependencies by dynamically weighting the contribution of all positions in the image. In our review, a pure Transformer (Pyramid Vision Transformer, PVT) was used by Viriyasaranon et al. (35). Hybrid models that combine CNN local feature extraction with Transformer global context were proposed by Liao et al. (36) (MVIT-MLKA) and Cao et al. (37) (PANDA, which uses a memory Transformer branch in the third stage for lesion subtype classification).

#### 2.4.5 Task-enhanced models with explicit clinical prior integration

These models are not purely data-driven; they explicitly encode clinical reasoning rules into their architecture.

Ramaekers et al. (29) used separate 3D U-Nets to segment the pancreatic duct and common bile duct. The resulting dilation maps were fed as additional input channels to the tumour detection network, encoding the clinical rule that “ductal dilation suggests malignancy.”

Degand et al. (38) (PANCANAI) employed an nnU-Net to segment the pancreas, lesions, and main pancreatic duct (MPD). Two independent logistic regression models then predict lesion presence and MPD dilatation; a positive decision is made if either criterion is met. This design directly exploits the clinical knowledge that MPD dilatation can precede visible tumour formation.

### 2.5 Exploratory analysis of traditional machine learning models

To comprehensively evaluate the diagnostic performance of deep learning models and compare them with traditional machine learning approaches, we performed an independent exploratory meta-analysis of studies that used only traditional machine learning algorithms (e.g., random forest, support vector machines, XGBoost) and otherwise met the inclusion criteria of the primary analysis. Owing to the limited number of included studies (*n* = 5) and the presence of a study with perfect diagnostic performance (zero false negatives), the bivariate random-effects model failed to converge. Therefore, we adopted a univariate random-effects model (restricted maximum likelihood, REML) to pool sensitivity and specificity separately. A continuity correction (adding 0.5 to each cell) was applied for studies with zero cells. Heterogeneity was assessed using the I^2^ statistic. This analysis is exploratory in nature, and the results should be interpreted with caution.

### 2.6 Quality assessment

The methodological quality of the eligible studies was independently assessed by two authors via the following tools: the Quality Assessment of Diagnostic Accuracy Studies 2 (QUADAS-2) (39), the Radiomics Quality Score (RQS) (40), the METhodological RadiomICs Score (METRICS) (41), and the Checklist for Artificial Intelligence in Medical Imaging (CLAIM) (42).

The QUADAS-2 tool is recommended for systematic reviews of diagnostic accuracy studies and evaluates four key domains: patient selection, index test, reference standard, and flow and timing. Each domain is assessed for risk of bias, and the first three domains are also evaluated for concerns regarding applicability.

The RQS, proposed by, is a tool designed to improve the methodological quality of radiomics research. It consists of 16 items covering the entire radiomic research workflow. The total RQS ranges from −8 to 36 points and can be converted to a percentage. Although widely adopted by the scientific community, the RQS has recognized limitations, including its failure to weight items according to their relative importance to overall study quality and the reported low reproducibility of its total and item-level scores (40, 43).

METRICS is a recently developed tool created by a large international consortium of experts, accessible at https://metricsscore.github.io/metrics/METRICS.html. It is user-friendly and specifically designed to enhance the methodological quality of radiomics research. The METRICS uniquely introduces expert opinion-based importance weights across 9 categories and 30 items via a transparent methodology. The overall quality of each study is categorized on the basis of its total score: 0 ≤ score < 20% as “very low”; 20 ≤ score < 40% as “low”; 40 ≤ score < 60% as “moderate”; 60 ≤ score < 80% as “good”; and 80 ≤ score ≤ 100% as “excellent.”

The CLAIM checklist is a reporting guideline specifically designed for AI applications in medical imaging. It comprises 42 items across six main sections: title/abstract, introduction, methods, results, discussion, and other information. This checklist aims to promote clear, transparent, and reproducible scientific communication for AI studies, providing detailed guidance tailored to the unique aspects of technologies such as deep learning. The key areas covered include study design, data preprocessing, model architecture, training procedures, performance evaluation, and model interpretability.

Any disagreements between the two assessing authors were resolved through discussion.

### 2.7 Statistical analysis

The results of the systematic review and meta-analysis evaluating deep learning-based diagnostic models for detecting pancreatic cancer on CT were analyzed on the basis of contingency tables (including true positives, false negatives, true negatives, and false positives) constructed for each included study. All the statistical analyses were performed via R software (version 4.5.1) and Stata (version 18.0; Stata Corporation LLC, College Station, USA) (44).

Forest plots were generated to visually present the pooled sensitivity and specificity estimates. A bivariate random-effects model, as described by Reitsma et al. (45) was employed to calculate the summary estimates for sensitivity, specificity, diagnostic odds ratio (DOR), and the area under the summary receiver operating characteristic (SROC) curve (46). The SROC curve, which synthesizes sensitivity and specificity values across different threshold settings from individual studies, was used for the overall assessment of diagnostic performance (45).

The area under the SROC curve (AUC) serves as a global measure of diagnostic accuracy and is commonly used to report the performance of deep learning detection models. The ROC curve was plotted with the true positive rate (sensitivity) on the y-axis and the false positive rate (1 - specificity) on the x-axis. Diagnostic performance on the basis of the AUC was categorized as follows: 0.90–1.00 (excellent), 0.80–0.90 (good), 0.70–0.80 (fair), 0.60–0.70 (poor), and 0.50–0.60 (failure).

Between-study heterogeneity was assessed via the inconsistency index (I^2^). The I^2^ values were interpreted as follows: 0–25% represented very low heterogeneity, 25–50% represented low heterogeneity, 50–75% represented moderate heterogeneity, and values exceeding 75% represented high heterogeneity. It should be noted that I^2^ measures the degree of variation in effect sizes across studies rather than serving as a direct assessment of study quality. High heterogeneity (e.g., I^2^ > 75% in our study) is common in diagnostic test accuracy meta-analyses and may arise from methodological diversity (e.g., differences in study design, CT parameters, or deep learning architectures) or clinical diversity (e.g., variations in population characteristics or disease stages). Importantly, high heterogeneity does not directly invalidate the pooled results. To explore potential sources of heterogeneity, we performed a prespecified subgroup analysis on prediagnostic scans and used a bivariate random-effects model with restricted maximum likelihood estimation to handle heterogeneity.

To further quantify heterogeneity, we additionally calculated three I^2^ estimates from the bivariate model: the conventional unadjusted I^2^ (Holling approach), the sample-size-adjusted I^2^ (Holling), and the Zhou–Dendukuri bivariate I^2^ which accounts for the correlation between sensitivity and false-positive rate (47). The Zhou–Dendukuri I^2^ is considered more valid for diagnostic test accuracy meta-analyses as it removes artefactual inflation caused by threshold effects and sample size differences. Threshold effects are inherently accommodated by the bivariate model, which estimates the correlation between logit (sensitivity) and logit (false-positive rate); a negative correlation supports the presence of a threshold effect.

Handling of zero cells. In the primary analysis of deep learning models, when any cell of the 2 × 2 contingency table (TP, FN, FP, or TN) contained a zero count, a continuity correction of 0.5 was added to all four cells of that study before calculating logit-transformed sensitivity, specificity, or diagnostic odds ratio. This correction prevents undefined values (e.g., division by zero) and is a standard conservative approach in diagnostic test accuracy meta-analyses. The robustness of this correction was verified by a sensitivity analysis excluding studies with zero cells, which yielded nearly identical pooled estimates (data not shown).

The R code used for all statistical analyses, including the bivariate model, meta-regression, leave-one-out analysis, and publication bias tests, is provided as Supplementary Code 1.

However, this reductionist strategy has important methodological limitations. By preferentially selecting the best-performing model within each study, the pooled estimates may reflect relatively optimistic estimates of diagnostic performance and may not fully capture within-study variability across evaluated models. A multilevel meta-analysis incorporating all reported models was considered but was not feasible because most primary studies did not provide complete 2 × 2 contingency table data for all evaluated models, and reporting formats were highly heterogeneous.

## 3 Results

### 3.1 Literature search

Using the predefined search strategies, 1,225 records were initially identified from the electronic databases (PubMed, *n* = 697; Web of Science, *n* = 494; Cochrane Library, *n* = 34). After removing 275 duplicate records, 950 records remained. Before title and abstract screening, 192 records were excluded because they were ineligible publication types (including books, review articles, systematic reviews, and meta-analyses), leaving 758 records for title and abstract screening. After screening, 701 records were excluded, and 57 full-text articles were assessed for eligibility. Following full-text review, 42 studies were excluded for the following reasons: MRI-based studies (*n =* 3), lack of internal or external validation (*n =* 25), insufficient data to construct a 2 × 2 contingency table (*n* = 9), and studies employing exclusively traditional machine learning models (*n =* 5) (48–52). Finally, 15 studies met the eligibility criteria and were included in the systematic review and meta-analysis (21–29, 33, 35–38, 53). All included studies were published in English. The study selection process is summarized in Figure 1.

**Figure 1:**
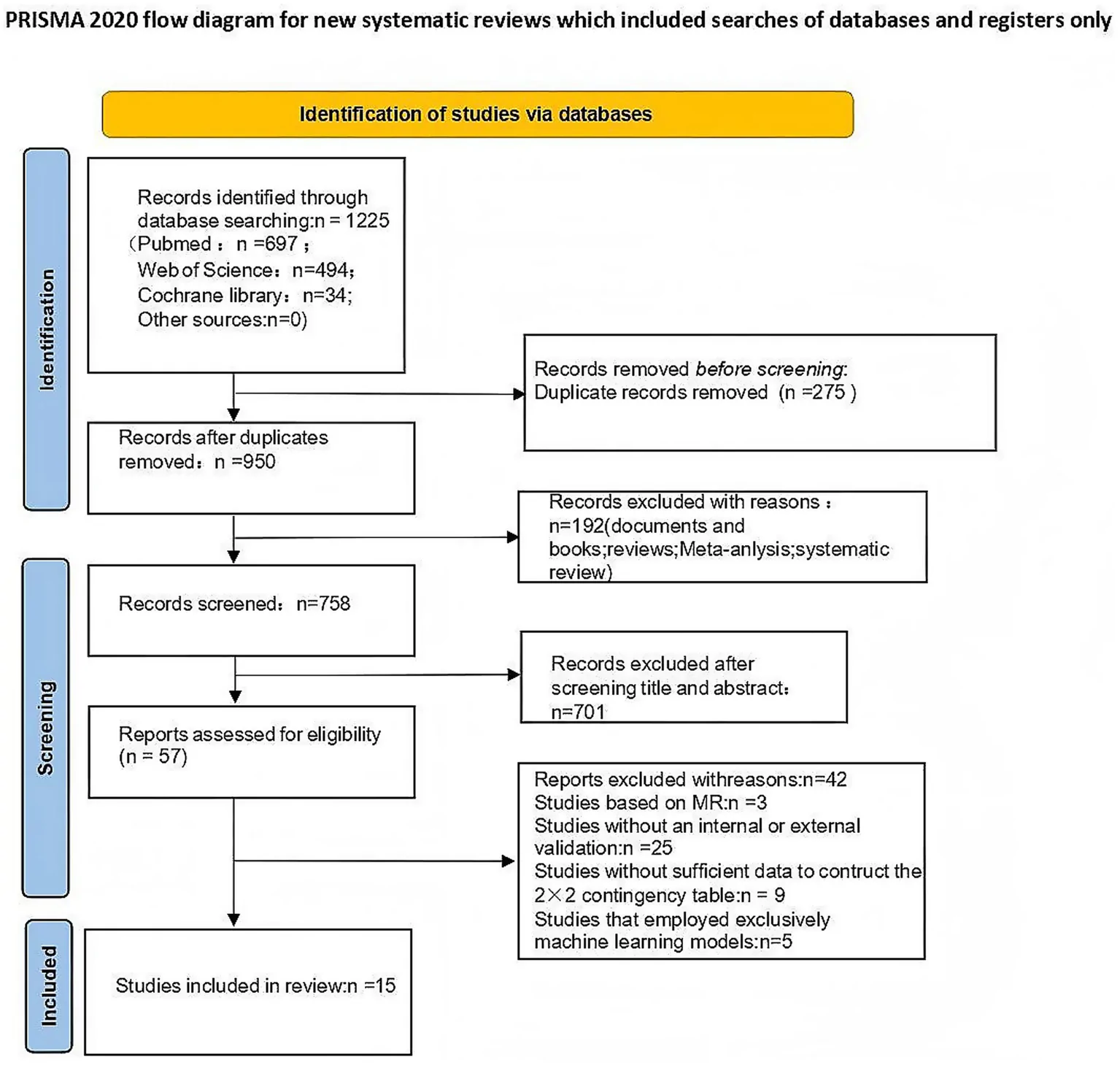
Flow diagram of the study selection process. A systematic search of PubMed, Web of Science, Cochrane Library, and IEEE up to September 11, 2025 identified 1,225 records. After removing 275 duplicates, 950 records remained. A further 192 records were excluded as ineligible publication types (e.g., books, reviews, systematic reviews, and meta-analyses), leaving 758 records for title and abstract screening. After screening, 701 records were excluded, and 57 full-text articles were assessed for eligibility. Full-text review led to the exclusion of 42 studies for the following reasons: MRI-based studies (*n* = 3), lack of internal or external validation (*n* = 25), insufficient data to construct 2 × 2 contingency tables (*n* = 9), and studies employing exclusively traditional machine learning models (*n* = 5). Finally, 15 studies met the eligibility criteria and were included in the quantitative meta-analysis.

### 3.2 Characteristics of the included studies

Among the 15 studies included in this meta-analysis and systematic review investigating the diagnostic performance of deep learning models for pancreatic cancer detection via CT, all were designed retrospectively, comprising both single-center (*n =* 8) and multicenter (*n =* 7) investigations. These studies originated from a diverse range of geographical regions, including China, the USA, South Korea, India, the Netherlands, Denmark, and Pakistan. Considerable variation was observed in sample sizes, ranging from hundreds to several thousand subjects. The majority of studies explicitly partitioned their data into training, internal test, and external validation sets to systematically evaluate model generalizability. The mean patient age was predominantly between 60 and 70 years, with the sex distribution being roughly balanced or showing a slight male predominance. All analyses were based on CT imaging, with the vast majority utilizing contrast-enhanced CT, and the portal venous phase served as the primary phase for analysis. The deep learning architectures employed were diverse, encompassing 3D-CNN, VGG, U-Net, ResNet, nnU-Net, and various innovative or ensemble models, such as the causality-inspired CNN, MVIT-MLKA, and DeepOptimalNet. The reference standard was highly consistent across studies, primarily on the basis of pathological diagnosis. Notably, several studies have explored the ability of models to identify cancer in “prediagnostic” images acquired months to years prior to clinical diagnosis, highlighting the significant potential of deep learning for early detection and warning of pancreatic cancer (see Table 1).

**Table 1:** Characteristics of included studies.

Authors and year of publication	Study location	Study design	Sample size	Age Mean±SD	Gender male:female	Tumor stage/clinical stage	Imaging modality	Contrast agent phase for analysis	Model	Reference standard
Korfiatis et al., 2023 (26)	USA	Retrospective, multi-center	Training-validation set *n* = 1776			T1:0	Contrast-enhanced CT	Portal venous phase	3D-CNN	Pathological diagnosis
			PDA *n* = 696	65.1 ± 10	M:f = 1.4:1	T2:155				
			Control group *n* = 1, 080	56.4 ± 16.2	m:f = 0.85:1	T3:186				
						T4:355				
			Internal test set *n* = 1, 238			T1:35				
			PDAC *n* = 409	66.6 ± 10	M:f = 1.2:1	T2:291				
			Control group *n* = 829	55.8 ± 15.8	M:f = 0.8:1	T3:60				
						T4:23				
			External test set *n* = 274			T1:25				
			PDAC *n* = 194			T2:133				
			Control group *n* = 80			T3:30				
						T4:26				
			Pre-diagnostic test set *n* = 233			Diagnosis 475 days prior (93–1, 082)				
	Taiwan, China	Retrospective, single-center	Training-validation set *n* = 551			T1:6	Contrast-enhanced CT	Portal venous phase	CNN (VGG)	Pathological diagnosis
			PDA *n* = 295	65.1 ± 12.2	m:f = 1.22:1	T2:97				
			control group *n* = 256			T3:53				
						T4:139				
			Local test set1 *n* = 139			T1:2				
			PDAC *n* = 75	63.9 ± 11.9	M:f = 1.13:1	T2:23				
			Control group *n* = 64			T3:6				
						T4:44				
			Local test set2 *n* = 189			T1:9				
			PDAC *n* = 101	65.9 ± 11.5	M:f = 1.27:1	T2:47				
			Control group *n* = 88			T3:15				
						T4:30				
			USA test set *n* = 363							
			PDAC *n* = 281							
			Control group *n* = 82							
Chen et al., 2023 (53)	Taiwan, China	Retrospective, single-center	Training-validation cohort *n* = 937			T1:23	Contrast-enhanced CT	Portal venous phase	Segmentation and Classification CNN	Pathological diagnosis
			PDAC *n* = 437	65 ± 12	M:f = 1.17:1	T2:175				
			Control group *n* = 536	54 ± 16	M:f = 1.04:1	T3:66				
						T4:173				
			Internal test set *n* = 256			T1:4				
			PDA *n* = 109	64 ± 12	M:f = 1.04:1	T2:46				
			Control group *n* = 147	55 ± 16	M:f = 1:1	T3:13				
			External test set *n* = 1, 473							
			PDAC *n* = 669							
			Control group *n* = 804							
Cao et al., 2023 (37)	Taiwan, China	Retrospective, single-center	Training-validation cohort *n* = 551			T1:6	CT		CNN (VGG)	Pathologicaldiagnosis orstandard clinical diagnosis
			PDAC *n* = 295	65.1 ± 12.2	m:f = 1.22:1	T2:97				
			Control group *n* = 256			T3:53				
						T4:139				
			Local test set1 *n* = 139			T1:2				
			PDAC *n* = 75	63.9 ± 11.9	M:f = 1.13:1	T2:23				
			Control group *n* = 64			T3:6				
						T4:44				
			Local test set2 *n* = 189			T1:9				
			PDAC *n* = 101	65.9 ± 11.5	M:f = 1.27:1	T2:47				
			Control group *n* = 88			T3:15				
						T4:30				
			USA test set *n* = 363							
			PDA *n* = 281							
			Control group *n* = 82							
			External multicenter							
			PDAC *n* = 2, 737							
			Control group *n* = 2, 600			pre-diagnosis				
			Real-world test set1							
			PDAC *n* = 44							
			control group *n* = 16, 420							
			Real-world test set2			Pre-diagnosis				
			PDAC *n* = 32							
			Control group *n* = 4, 110							
Qu et al. 2024 (27)	China	Retrospective, multi-center	Cross-validation set *n* = 442	Not reported		Clinical diagnosis 3–36 months prior	Contrast-enhanced CT	Arterial and venou phase	Causality-inspired 3D CNN	Pathological diagnosis
			PDAC *n* = 223							
			Control group *n* = 219							
			Independent test set1 *n* = 264							
			PDAC *n* = 107							
			Control group *n* = 157							
			Independent test set2 *n* = 155							
			PDAC *n* = 88							
			Control group *n* = 67							
			Independent test set *n* = 363							
			PDAC *n* = 281							
			Control group *n* = 82							
Ma et al., 2020 (22)	China	Retrospective, single-center	Control group *n* = 190	63.8 ± 8.7	m:f = 1.07:1		Contrast-enhanced CT			
			PDAC *n* = 222	61.0 ± 12.3	m:f = 1.27:1	3.5 (2.7–4.3)			CNN (VGG)	Pathological diagnosis
Si et al., 2021 (25)	China	Retrospective, multi-center	Training set *n* = 319	63.3	M:f = 1.95:1		Contrast-enhanced CT		ResNet18, U-Net32, ResNet34	Pathological diagnosis
			PDAC *n* = 209							
			Control group *n* = 35							
			Test set *n* = 347	61.8	M:f = 1.65:1					
			PDAC *n* = 170							
			Control group *n* = 82							
Park et al., 2023 (28)	South Korea	Retrospective, multi-center	Training set *n* = 852	60	m:f = 1.18:1	3.1 (0.5–8.8)		Portal venous phase	3D nnU-Net	Pathological diagnosis
			PDAC *n* = 284				Contrast-enhanced CT			
			Control group *n* = 568							
			Test set1 *n* = 603	18–82	m:f = 1.66:1	3.0 (0.8–7.6)				
			PDAC *n* = 45							
			Control group *n* = 558							
			Test set2 *n* = 589	18–99	m:f = 1.39:1	2.4 (0.9–5.0)				
			PDAC *n* = 48							
			Control group *n* = 541							
			PDAC *n* = 30	66.9	m:f = 1.5:1					
Ramaekers et al., 2024 (29)	Netherlands	Retrospective, single-center	Full cohort *n* = 197				Contrast-enhanced CT	Portal venous phase	3D U-Net	
			Control group *n* = 98	71.2 ± 8.1	M:f = 1.11:1					
			PDAC *n* = 99	74.9 ± 7.5	m:f = 4.16:1	T1:21				
						T2:55				
						T3:20				
						T4:3				
Viriyasaranon et al., 2023 (35)	South Korea	Retrospective, multi-center	External validation set *n* = 361	58.9 ± 13.4			Contrast-enhanced CT	Portal venous and pancreatic phase	ShuffleNet + PS	Pathological diagnosis
			PDAC *n* = 281							
			Control group *n* = 80							
Mandal et al., 2024 (33)	India	Retrospective, multi-center	Test set *n* = 1, 577				Contrast-enhanced CT	Portal venous phase	nnU-Net + MIL + NN	Pathological diagnosis
			control group *n* = 179							
			PDAC *n* = 179							
Liao et al., 2025 (36)	China	Retrospective, multi-center	Total *n* = 864	56.4 ± 12.9	m:f = 1:1.05		Contrast-enhanced CT	Portal venous phase	MVIT-MLKA	Pathological diagnosis
			Training: *n* = 513							
			Internal testing cohort: *n* = 129							
			External validation: *n* = 222							
Thanya et al., 2025 (24)	India	Retrospective, single-center	Training-validation set *n* = 690 PDAC *n* = 370, Control *n* = 320	PDAC: 68 ± 13.5 Control: 69 ± 14.5	PDAC: m:f = 1.4:1		CT	Not Specified	DeepOptimalNet	Pathological diagnosis
			Test set *n* = 189		Control: m:f = 1.2:1	pre-diagnosis				
			PDAC *n* = 101, Control *n* = 88							
Degand et al., 2025 (38)	Denmark	Retrospective, multi-center	Total Patients: *n* = 1, 083 Total CT Scans: *n* = 1, 220	69 ± 11	m:f = 1.13:1		Contrast-enhanced CT	Primarily Portal venous phase (90%), also included Arterial (6%) and Delayed (4%) phases	PANCANAI	Histopathological diagnosis
			Concurrent Diagnosis Group: *n* = 1, 022 scans			within 2 months				
			Pre-diagnosis Group: *n* = 198 scans			7 months prior				
			This study is an external validation the model was previously trained on 2, 134 scans.							
Nadeem et al., 2025 (23)	Pakistan	Retrospective, single-center	Total: 1411 CT images	Not reported	Not reported	Not reported	Contrast-enhanced CT	Not specified	Four-stage CAD system	Histopathological diagnosis
			PDAC/Tumor: 765 images							
			Control: 646 images							
			Test set: 282 images PDAC = 153, Control = 129							

All included studies were retrospective (eight single-center and seven multicenter) and used contrast-enhanced CT with histopathological diagnosis as the reference standard. Sample sizes ranged from approximately 200 to over 10, 000 participants, with mean patient age typically between 60 and 70 years. Deep learning architectures included 3D-CNN, ResNet, U-Net, nnU-Net, hybrid CNN–Transformer models, and task-enhanced models incorporating clinical priors. Four studies evaluated prediagnostic CT scans acquired 3–36 months before diagnosis. Key observation: Substantial variability in study design, population, and model architecture may contribute to the heterogeneity observed in pooled diagnostic performance.

### 3.3 Methodological quality

The methodological quality of the studies according to the QUADAS-2 assessment is illustrated in Figures 2A,B. Regarding the risk of bias, the domains of “Patient Selection” and “Flow and Timing” presented the most significant concerns, with a substantial proportion of studies rated as having a high or unclear risk of bias. This is primarily attributable to the retrospective nature of the included studies and potential nonconsecutive patient enrollment. In contrast, the “Index Test” (i.e., the interpretation by the deep learning models) and “Reference Standard” (primarily based on pathological diagnosis) domains were predominantly assessed as low risk. This assessment stems from the typical blinding procedures employed, where the index test interpretation was conducted without knowledge of the reference standard results, and vice versa. Concerning applicability, all domains were judged as low concern for the vast majority of studies, indicating a strong alignment between the review’s research question and the patient populations, index tests, and reference standards utilized in the primary studies. In summary, while the risk of bias in certain methodological domains warrants cautious interpretation of the findings, the low concerns regarding applicability reinforce the relevance and validity of the synthesized results within the clinical context of pancreatic cancer detection on CT scans.

**Figure 2:**
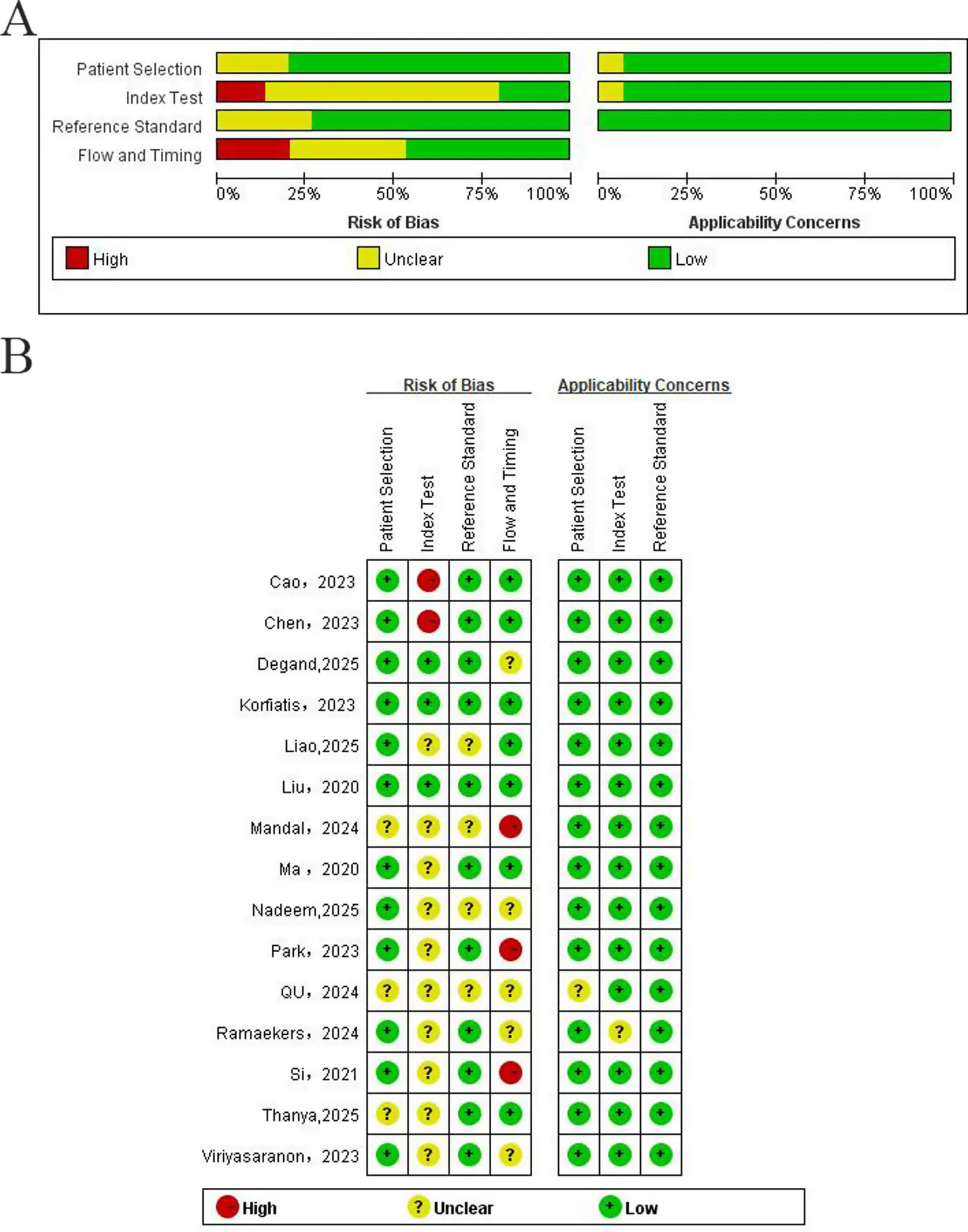
**(A)** QUADAS-2 summary of risk of bias and applicability concerns across included studies. The bar chart summarizes the proportion of studies rated as low, unclear, or high risk of bias across four domains (patient selection, index test, reference standard, and flow and timing). The x-axis represents the QUADAS-2 domains, and the y-axis represents the percentage of studies. The right panel shows applicability concerns using the same domains. Key observations: Most studies demonstrate low risk of bias for the index test (deep learning model) and reference standard (histopathological diagnosis). However, the patient selection and flow/timing domains show a high or unclear risk of bias in more than 50% of studies, largely due to retrospective designs and non-consecutive sampling. Applicability concerns are consistently low, indicating that the included studies are generally relevant to the review question. Interpretation: These patterns suggest that selection bias and study design limitations may contribute to the substantial heterogeneity observed in pooled diagnostic estimates. **(B)** Individual study QUADAS-2 assessment matrix. Each row represents an individual study, and each column corresponds to one QUADAS-2 domain (patient selection, index test, reference standard, and flow and timing). Green circles indicate low risk or low concern, yellow circles indicate unclear risk, and red circles indicate high risk. Key findings: most studies show low risk of bias for the index test and reference standard domains, while several studies exhibit unclear or high risk in patient selection and flow/timing domains. All studies demonstrate low applicability concern for the reference standard. Interpretation: The predominance of unclear or high risk in selection-related domains reflects common limitations of retrospective study designs and may impact the generalizability of reported model performance.

The methodological quality of the included deep learning-based studies was evaluated using the Radiomics Quality Score (RQS), which has a maximum possible score of 36. The total RQS scores ranged from 6 to 24, with a mean score of 16.4, corresponding to 45.6% of the maximum achievable score (Table 2). Overall, the methodological quality of the included studies was moderate. Ramaekers et al. (29) and Cao et al. (37) achieved the highest RQS scores (24 and 23, respectively), indicating relatively strong adherence to radiomics quality standards. Common strengths included image protocol quality, discrimination statistics, comparison with the reference standard, and validation procedures. However, several important methodological deficiencies were consistently identified across studies, including the absence of phantom studies, limited investigation of biological correlates, lack of cost-effectiveness analyses, and insufficient adherence to open science practices. In particular, the limited availability of publicly shared source code and imaging data reduced the reproducibility and external validation potential of the proposed deep learning models. Overall, although the included studies demonstrated acceptable methodological quality in several key domains, substantial improvements in biological validation, economic evaluation, transparency, and data sharing are still needed to facilitate the clinical translation of deep learning models for pancreatic cancer detection.

**Table 2:** Radiomics quality score (RQS) assessment of included studies.

Study criteria	Korfiatis et al., 2023 (26)	Liu, 2020 (21)	Chen et al., 2023 (53)	Cao et al., 2023 (37)	Qu et al. 2024 (27)	Ma, 2020	Si et al., 2021 (25)	Park et al., 2023 (28)	Ramaekers et al., 2024 (29)	Viriyasaranon et al., 2023 (35)	Mandal et al., 2024 (33)	Liao et al., 2025 (36)	Thanya et al., 2025 (24)	Degand et al., 2025 (38)	Nadeem et al., 2025 (23)	Average score
Image protocol quality (+1 or +2)	1	1	1	2	1	2	1	2	2	2	2	2	1	2	1	1.53
Multiple segmentation (+1)	0	1	0	0	0	1	1	0	1	0	0	1	2	2	0	0.6
Phantom study (+1)	0	0	0	0	0	1	0	0	1	0	0	0	0	0	2	0.27
Imaging at multiple time points (+1)	0	0	0	0	1	1	0	0	1	0	0	0	0	2	2	0.47
Feature reduction or adjustment for multiple testing (−3 or +3)	3	0	3	3	-3	0	3	0	0	1	3	3	1	2	2	1.4
Multivariable analysis (+1)	1	0	1	0	0	1	0	0	1	0	0	0	0	1	1	0.4
Biological correlates (+1)	1	0	1	0	0	1	0	0	1	0	0	0	0	0	0	0.27
Cut-off analysis (+1)	0	0	0	1	0	1	0	1	1	0	1	1	1	1	2	0.67
Discrimination statistics (+1 or +2)	1	1	1	2	1	2	1	2	2	1	2	2	2	2	2	1.6
Calibration statistics (+1 or +2)	0	0	0	1	0	2	0	1	2	0	1	2	0	0	0	0.6
Prospective study (+7)	0	0	0	7	0	0	0	7	0	0	7	1	0	0	0	1.47
Validation (−5 to +5)	3	2	3	3	2	2	3	3	2	2	-5	3	4	5	2	2.27
Comparison to ‘gold standard’ (+2)	2	2	2	2	0	2	2	2	2	0	2	2	2	2	0	1.6
Potential clinical application (+2)	2	2	2	2	1	2	3	2	2	1	2	2	1	2	1	1.8
Cost-effectiveness analysis (+1)	1	0	1	0	0	1	0	0	1	0	0	0	0	0	1	0.33
Open science and data (+1 to +4)	1	0	1	3	0	4	1	3	4	1	3	1	0	1	0	1.53
Total score (−8 to +36)	15	9	13	23	6	23	14	20	24	9	18	20	14	22	16	16.4

RQS scores ranged from 6 to 24 (maximum 36), with a converted mean of 45.6%. Strengths included image protocol quality and discrimination statistics, while common limitations involved lack of phantom studies, biological correlates, cost-effectiveness analyses, and open science practices. Interpretation: These limitations may reduce reproducibility and hinder translation of deep learning models into clinical practice.

On the basis of the METhodological RadiomICs Score (METRICS) assessment, the overall methodological quality of the included studies yielded an average score of 62.21% ± 11.43%. According to the predefined quality categories, 53.3% (8/15) of the studies were rated as “good,” 40.0% (6/15) as “moderate,” and only one study (6.7%) was categorized as “low.” No study achieved an “excellent” rating. The METRICS-based item-level evaluation identified three common methodological deficiencies across the 15 included studies. First, regarding image preprocessing and feature extraction: although all 15 studies appropriately described their image preprocessing techniques, the vast majority (12/15, 80%) failed to transparently report feature extraction parameters or provide a default configuration statement. Critical details such as convolutional kernel sizes, stride, padding, and pooling layer configurations were frequently omitted, limiting the reproducibility of the models. Second, concerning feature stability analysis: most studies did not systematically assess the robustness of the extracted features. Specifically, 13 studies (86.7%) failed to remove non-robust features, none removed redundant features, and 8 studies (53.3%) did not demonstrate the appropriateness of feature dimensionality relative to sample size, substantially increasing the risk of overfitting and poor generalizability. Third, in terms of open science practices: the availability of research outputs was severely limited. Only 2 studies (13.3%) made their raw or preprocessed data publicly available, only 3 studies (20%) provided source code, and none of the studies (0/15) shared trained model weights. This fundamentally hinders external validation, replication, and clinical translation. These findings indicate that although the general methodological rigor of current radiomics research is acceptable (mean METRICS score 62.21%), there is a clear need for improvements in transparent reporting of feature extraction parameters, routine implementation of feature stability analyses, and adherence to open science principles (see Table 3).

**Table 3:** Methodological quality assessment of included studies using the METhodological RadiomICs Score (METRICS).

Categories	No.	Korfiatis et al., 2023 (26)	Liu, 2020 (21)	Chen et al., 2023 (53)	Cao et al., 2023 (37)	Qu et al. 2024 (27)	Ma, 2020	Si et al., 2021 (25)	Park et al., 2023 (28)	Ramaekers et al., 2024 (29)	Viriyasaranon et al., 2023 (35)	Mandal et al., 2024 (33)	Liao et al., 2025 (36)	Thanya et al., 2025 (24)	Degand et al., 2025 (38)	Nadeem et al., 2025 (23)
Study design	#1	No	Yes	No	Yes	Yes	Yes	Yes	Yes	0.0368	No	No	Yes	No	No	No
	#2	Yes	Yes	Yes	Yes	Yes	Yes	Yes	Yes	Yes	Yes	Yes	Yes	Yes	Yes	Yes
	#3	Yes	Yes	Yes	Yes	Yes	Yes	Yes	Yes	Yes	No	Yes	Yes	Yes	Yes	No
Imaging data	#4	No	No	Yes	Yes	Yes	No	No	Yes	No	No	Yes	Yes	Yes	Yes	No
	#5	Yes	Yes	Yes	Yes	Yes	Yes	Yes	Yes	Yes	Yes	Yes	Yes	Yes	Yes	Yes
	#6	Yes	Yes	Yes	Yes	No	Yes	Yes	Yes	Yes	Yes	Yes	Yes	No	Yes	No
	#7	No	Yes	No	Yes	No	Yes	Yes	Yes	No	No	No	Yes	Yes	Yes	No
Segmentation^a^	#8	Yes	Yes	Yes	Yes	No	Yes	Yes	Yes	Yes	Yes	Yes	Yes	Yes	Yes	Yes
	#9	No	No	No	Yes	No	No	Yes	Yes	No	No	No	No	No	Yes	No
	#10	No	No	No	Yes	No	No	Yes	Yes	No	No	No	No	No	Yes	No
Image processing and feature extraction	#11	Yes	Yes	Yes	Yes	Yes	Yes	Yes	Yes	Yes	Yes	Yes	Yes	Yes	Yes	Yes
	#12	No	No	No	No	Yes	No	No	No	Yes	No	Yes	No	No	No	No
	#13	No	No	No	No	No	No	No	No	No	No	Yes	No	No	Yes	Yes
Feature processing	#14	No	No	No	No	Yes	No	No	No	No	No	Yes	No	No	No	No
	#15	No	No	No	No	No	No	No	No	No	No	No	No	No	No	No
	#16	No	Yes	No	Yes	No	Yes	Yes	Yes	No	No	Yes	No	No	Yes	No
	#17	No	Yes	No	Yes	No	Yes	Yes	Yes	No	No	No	No	No	Yes	No
Preparation for modeling	#18	Yes	Yes	Yes	Yes	Yes	Yes	Yes	Yes	Yes	Yes	Yes	Yes	Yes	Yes	Yes
	#19	No	No	No	No	Yes	No	No	No	No	No	No	No	No	No	No
Metrics and comparison	#20	Yes	Yes	Yes	Yes	Yes	Yes	Yes	Yes	Yes	Yes	Yes	Yes	Yes	Yes	Yes
	#21	No	No	No	No	No	No	No	No	No	No	No	Yes	Yes	Yes	No
	#22	No	No	No	No	No	No	No	No	No	No	No	Yes	No	No	No
	#23	No	No	No	No	No	No	No	No	No	No	No	No	No	No	No
	#24	No	Yes	No	Yes	No	Yes	Yes	Yes	No	No	No	Yes	No	Yes	No
	#25	No	No	No	No	No	No	No	No	No	No	No	Yes	No	No	No
Testing	#26	Yes	Yes	Yes	Yes	Yes	Yes	Yes	Yes	Yes	Yes	Yes	Yes	Yes	Yes	Yes
	#27	Yes	Yes	Yes	Yes	Yes	No	Yes	Yes	Yes	Yes	No	Yes	Yes	Yes	No
Open science	#28	No	No	No	No	No	No	No	No	No	No	No	No	No	Yes	Yes
	#29	No	No	Yes	No	No	No	No	No	Yes	Yes	No	No	No	No	No
	#30	No	No	No	No	No	No	No	No	No	No	No	No	No	No	No
Total METRICS score (should be given as percentage)		54.18%	68.71%	59.31%	76.46%	58.01%	61.22%	72.08%	76.46%	59.53%	43.55%	63.33%	73.95%	59.44%	68.93%	38.02%
Quality category^g^		Moderate	Good	Moderate	Good	Moderate	Good	Good	Good	Moderate	Moderate	Good	Good	Moderate	Good	Low

The mean METRICS score was 62.21%. Eight studies were rated “good,” six “moderate,” and one “low.” Common deficiencies included incomplete reporting of feature extraction parameters and lack of data/code sharing. Interpretation: Limited transparency and reproducibility may hinder independent validation and reduce confidence in generalizability of reported model performance.

The reporting quality of the included studies was evaluated via the Checklist for Artificial Intelligence in Medical Imaging (CLAIM), which comprises 42 items. The evaluation revealed variable adherence to reporting standards across studies, with total scores ranging from 17−−34.5. The studies by Cao et al. (37) and Korfiatis et al. (26) demonstrated the highest reporting quality, with scores of 34.5 and 33, respectively. In contrast, the study by Si et al. (25) reported the lowest overall adherence, with a score of 17. A common observation was that many studies had a substantial number of partially fulfilled items, particularly in sections detailing data handling procedures, model development processes, and technical specifications. This pattern indicates that although most studies address fundamental reporting elements, the essential methodological details required for reproducibility are often insufficiently documented. Frequent reporting shortcomings were identified in the specification of data preprocessing steps, the rationale for hyperparameter selection, and the availability of code and trained models. In summary, while the included studies generally covered key aspects of AI research, significant improvements are needed in the granularity and transparency of methodological reporting to fully align with CLAIM guidelines and facilitate both replication and clinical translation.

The detailed CLAIM scores for the included studies are presented in Table 4.

**Table 4:** CLAIM (Checklist for artificial intelligence in medical imaging) assessment.

CLAIM assessment items	Korfiatis et al., 2023 (26)	Liu, 2020 (21)	Chen et al., 2023 (53)	Cao et al., 2023 (37)	Qu et al. 2024 (27)	Ma et al., 2020 (22)	Si et al., 2021 (25)	Park et al., 2023 (28)	Ramaekers et al., 2024 (29)	Viriyasaranon et al., 2023 (35)	Mandal et al., 2024 (33)	Liao et al., 2025 (36)	Thanya et al., 2025 (24)	Degand et al., 2025 (38)	Nadeem et al., 2025 (23)
Total projects	42	43	44	45	46	47	48	49	50	51	52	53	54	55	56
Compliant projects	30	26	20	30	12	12	4	24	21	18	15	27	20	11	12
Partially compliant projects	6	10	21	9	17	17	26	9	10	14	17	10	4	19	19
Non-compliant projects	6	5	1	2	13	13	11	9	11	10	10	4	12	8	7
Not applicable projects	0	1	0	1	0	0	1	0	0	0	0	0	6	4	4
Overall score	33	31	30.5	34.5	20.5	20.5	17	28.5	26	25	23.5	32	22	20.5	21.5

Scores ranged from 17 to 34.5 out of 42, with variability across studies. Lower compliance was observed in areas such as preprocessing, hyperparameter tuning, and code availability. Interpretation: Inconsistent adherence to reporting standards may contribute to variability in study transparency and comparability across the literature.

Based on the core requirements of QUADAS-2, METRICS, CLAIM, and RQS, we propose the following criteria for a primary study to be qualified as having “excellent” methodological quality.

#### 3.3.1 Study design

Prospective or high-quality retrospective multicenter design with consecutive case enrollment, blinded interpretation of the reference standard (histopathology), and pre-registration of the study protocol (if applicable).

#### 3.3.2 Technical rigor

Complete reporting of CT acquisition parameters and preprocessing steps; use of an independent internal test set and at least one external validation set; patient-level data partitioning. For handcrafted feature-based studies, the feature-to-sample size ratio should be appropriate; for end-to-end deep learning, the relationship between model complexity and dataset size should be justified.

#### 3.3.3 Model detail description

Detailed description of the model architecture, hyperparameters, and training process.

#### 3.3.4 Comprehensive evaluation

Reporting of the area under the curve (AUC), sensitivity, specificity, and their 95% confidence intervals, along with a calibration assessment (e.g., calibration curve) to evaluate model fit.

#### 3.3.5 Open science and reproducibility

Clear indication of deep learning in the title or abstract with a structured summary; provision of a study population flow diagram; and public sharing of at least one of the following: code, de-identified data, or model weights.

The results of Deeks’ funnel plot asymmetry test (*p* = 0.49) indicate no significant publication bias in the present meta-analysis, suggesting that the body of included studies demonstrates satisfactory completeness and that the pooled results are less susceptible to bias from missing smaller studies. This finding enhances the credibility of the synthesized evidence.

Deeks’ funnel plot is presented in Supplementary Figure 1.

### 3.4 Diagnostic accuracy of deep learning models

Forest plots of the sensitivity and specificity of the included studies are presented in Figures 3A,B. The forest plots demonstrate the diagnostic performance of the deep learning models for detecting pancreatic cancer on CT images. The pooled results under the random effects model revealed a high overall sensitivity of 0.92 (95% CI, 0.88–0.94) and specificity of 0.96 (95% CI, 0.92–0.98). The sensitivity estimates across individual studies were relatively consistent, mostly ranging between 0.85 and 0.95, whereas specificity exhibited greater variation, with values ranging from 0.70 to 1.00. Notably, significant heterogeneity was observed among the studies, with I^2^ values of 87.4% for sensitivity and 95.9% for specificity (both *p* < 0.0001), indicating substantial variations possibly attributable to differences in study design, population characteristics, or methodological approaches. Despite this heterogeneity, the combined results affirm that deep learning models achieve high diagnostic accuracy in detecting pancreatic cancer, effectively identifying true positives while maintaining a strong ability to discriminate true negatives. When applying the Zhou–Dendukuri bivariate I^2^, which adjusts for the correlation between sensitivity and specificity, the overall I^2^ was 37.1%, indicating moderate heterogeneity. The Holling sample-size-adjusted I^2^ was 1.8–2.9%, and the unadjusted Holling I^2^ ranged from 75.7 to 93.8%. These results demonstrate that the high conventional I^2^ values are largely driven by differences in sample size and threshold effects rather than by genuine inconsistency in deep learning performance. The estimated correlation between logit (sensitivity) and logit (false-positive rate) from the bivariate model was *ρ* = −0.419, consistent with the presence of a threshold effect.

**Figure 3:**
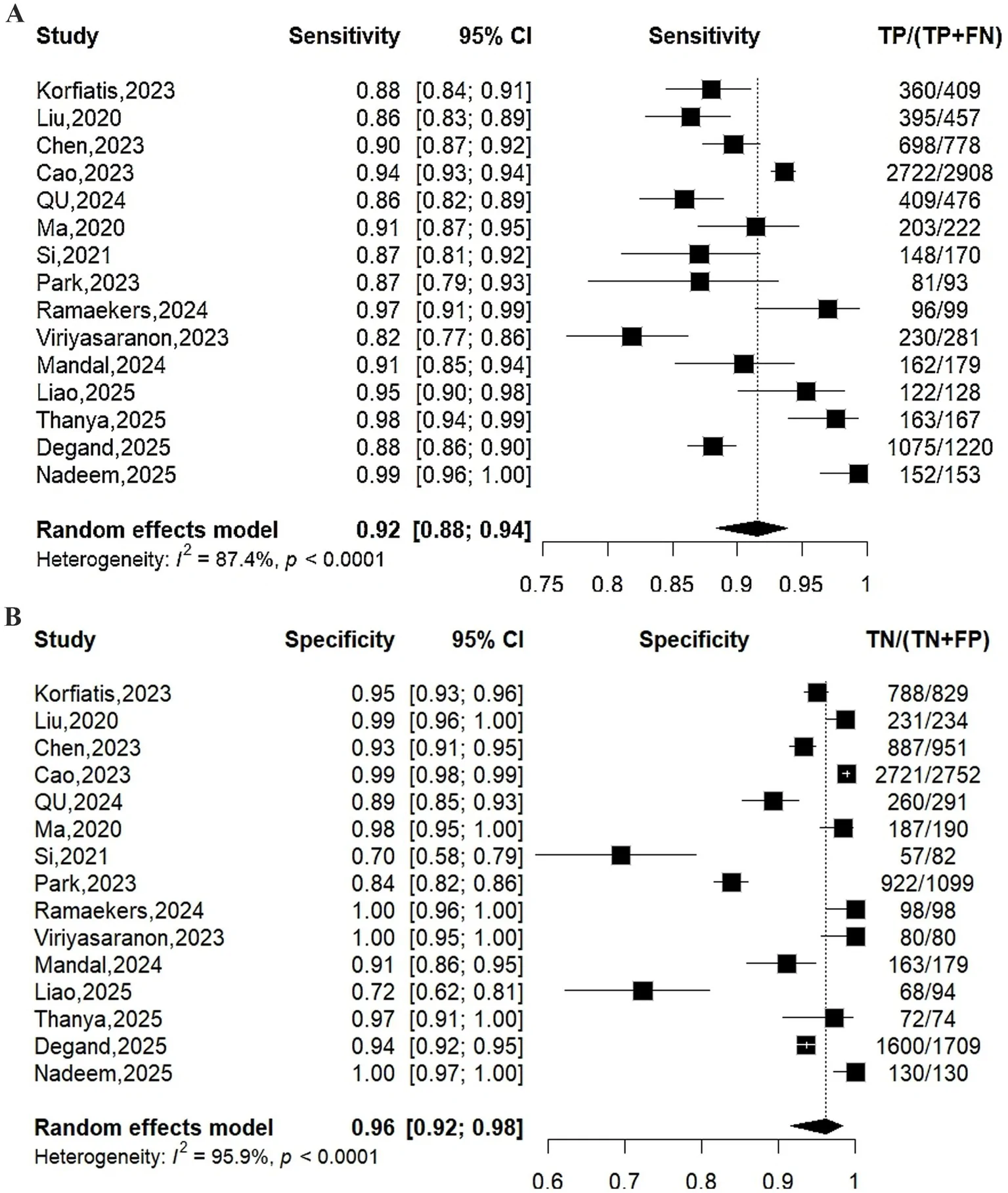
**(A)** Forest plot of sensitivity for deep learning models detecting pancreatic ductal adenocarcinoma on CT. Each square represents an individual study’s sensitivity estimate, with horizontal lines indicating 95% confidence intervals; square size is proportional to the study’s weight in the random-effects model. The x-axis represents sensitivity values, and the y-axis lists the included studies. The pooled sensitivity is 0.92 (95% CI: 0.88–0.94). Heterogeneity is substantial (*I*^2^ = 87.4%, *p* < 0.0001). Key takeaway: Despite high heterogeneity, deep learning models consistently demonstrate strong sensitivity, suggesting robust detection capability across diverse datasets, although variability highlights the need for standardized evaluation and external validation. **(B)** Forest plot of specificity for deep learning models detecting pancreatic ductal adenocarcinoma on CT. Each square represents an individual study’s specificity estimate, with horizontal lines indicating 95% confidence intervals; square size is proportional to the study’s weight in the random-effects model. The *x-ax*is represents specificity values, and the y-axis lists the included studies. The pooled specificity is 0.96 (95% CI: 0.92–0.98). Heterogeneity is substantial (*I*^2^ = 95.9%, *p* < 0.0001). Key takeaway: Deep learning models generally achieve excellent specificity, indicating strong control of false positives; however, variability across studies suggests sensitivity to dataset characteristics and model implementation.

The SROC curve (see Figure 4) demonstrated strong discriminative ability, with an area under the curve of 0.97 (95% CI: 0.95–0.98). This finding indicates high diagnostic performance and strong potential for accurate clinical detection of the target condition.

**Figure 4:**
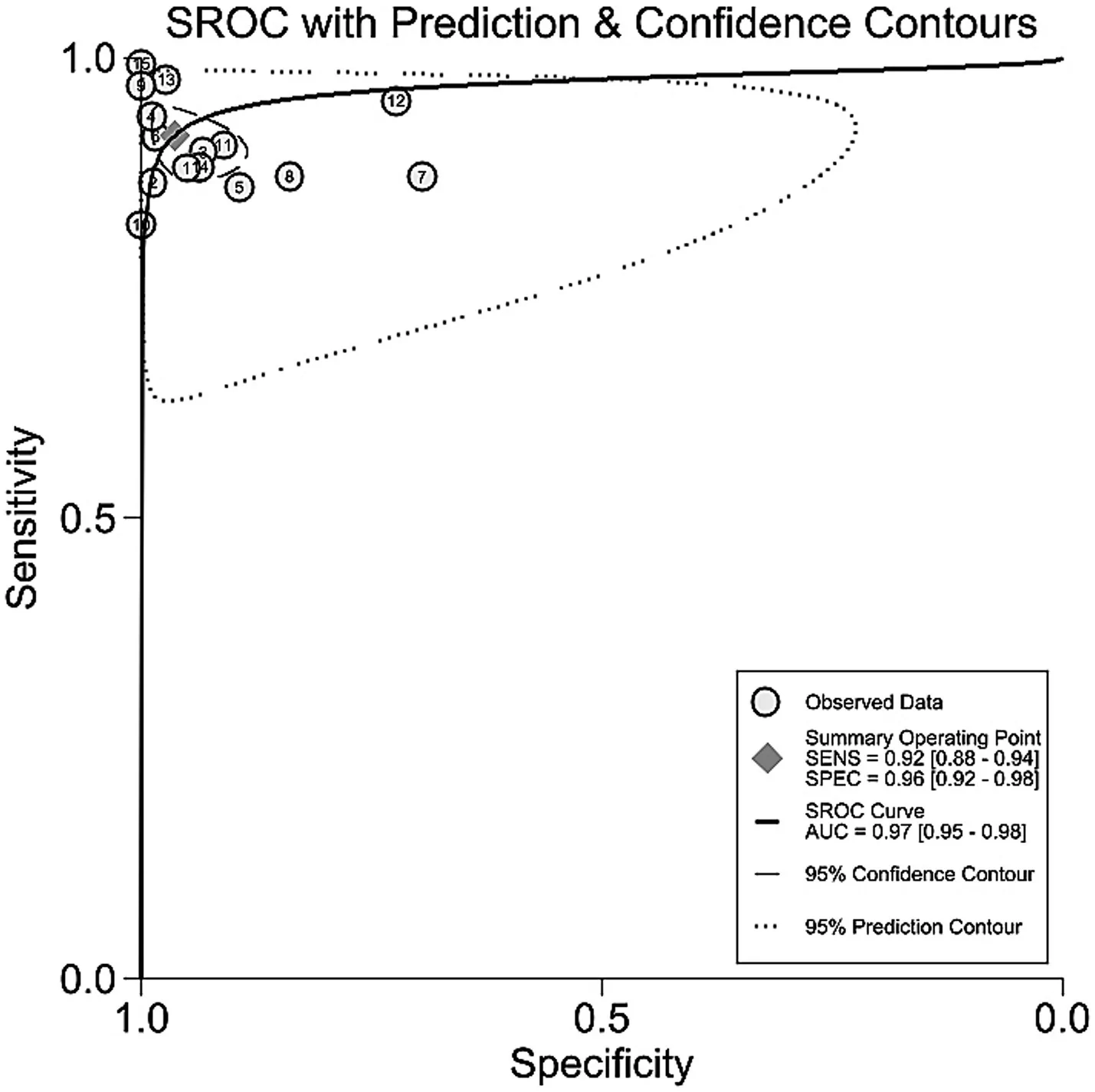
Summary receiver operating characteristic (SROC) curve of deep learning models for diagnosing PDAC on CT. Each solid circle represents an individual study’s paired sensitivity and specificity, plotted as (1 – specificity, sensitivity). The x-axis represents the false positive rate (1 – specificity), and the y-axis represents sensitivity. The summary operating point (diamond) reflects pooled estimates. The area under the curve is 0.97 (95% CI: 0.95–0.98). The dashed ellipse indicates the 95% confidence region. Key takeaway: The curve lies close to the upper-left corner, indicating high overall diagnostic accuracy; however, dispersion of study points reflects underlying heterogeneity and emphasizes the need for validation across populations.

The analysis of the preclinical diagnosis subgroup demonstrated that the deep learning model achieved a pooled sensitivity of 0.73 (95% CI: 0.69–0.77) and a pooled specificity of 0.92 (95% CI: 0.75–0.98), based on a bivariate random-effects model. Heterogeneity was quantified using the Zhou–Dendukuri bivariate I^2^ approach, which yielded a combined I^2^ of 0.4% (*p* < 0.0001), indicating very low between-study heterogeneity for the joint model. This finding indicates substantial variation in diagnostic performance across the included studies, potentially attributable to differences in study design, population characteristics, or diagnostic criteria. Thus, the pooled results should be interpreted with caution. Nonetheless, these findings provide preliminary evidence that deep learning models hold substantial promise for the preclinical diagnosis of pancreatic cancer. Forest plots of the sensitivity and specificity of the early-stage diagnostic subgroup are presented in Figures 5A,B.

**Figure 5:**
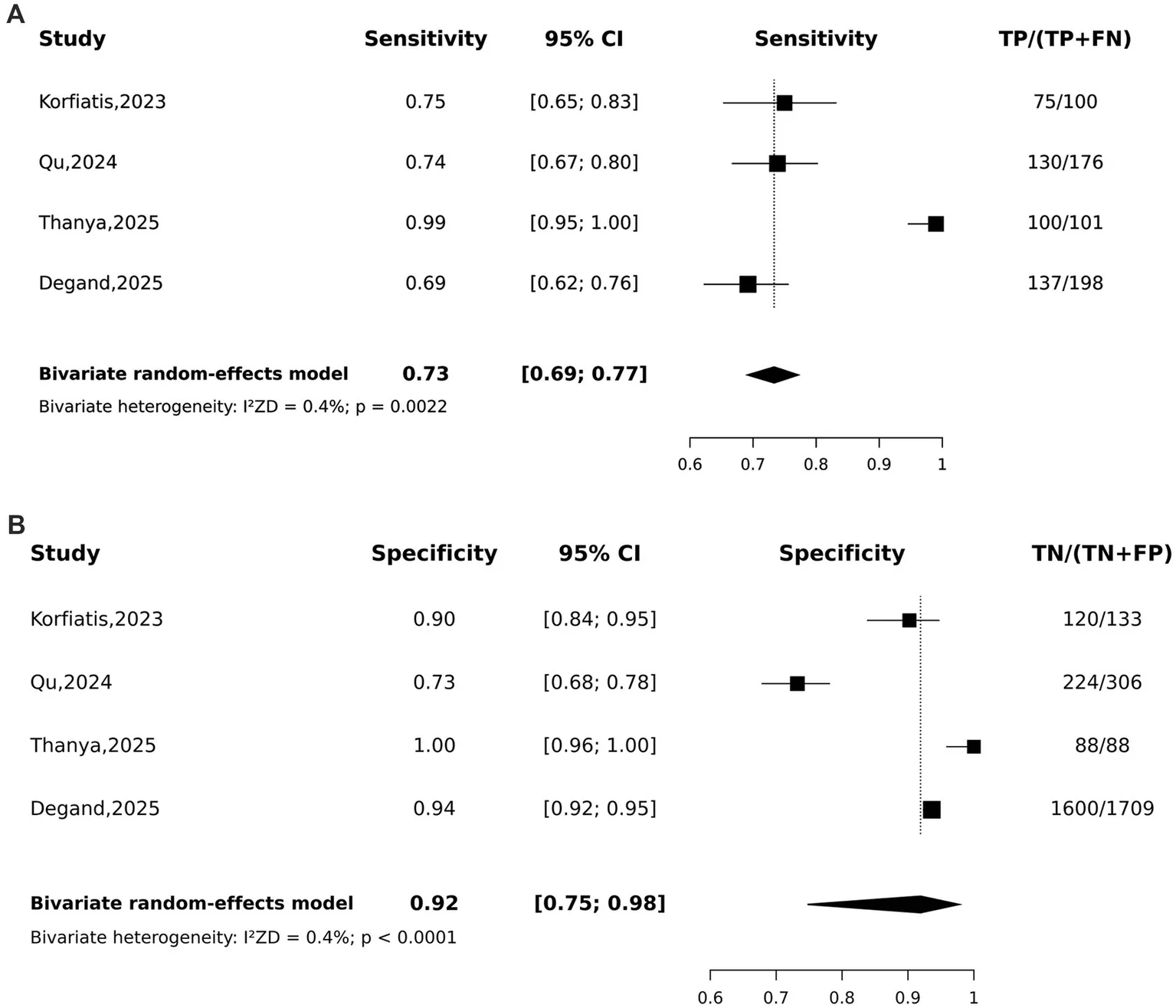
**(A)** Forest plot of sensitivity for the pre-diagnosis subgroup (CT scans acquired 3–36 months before clinical diagnosis). Each square represents an individual study’s sensitivity estimate, with horizontal lines indicating 95% confidence intervals; square size is proportional to study weight. The *x*-axis represents sensitivity values, and the *y*-axis lists the included studies. The pooled sensitivity was 0.73 (95% CI: 0.69–0.77), based on a bivariate random-effects model. Key takeaway: While promising, the wide confidence interval and extreme heterogeneity highlight uncertainty in performance and indicate that further studies are required to establish the robustness and clinical reliability of prediagnostic detection. **(B)** Forest plot of specificity for the pre-diagnosis subgroup. Each square represents an individual study’s specificity estimate, with horizontal lines indicating 95% confidence intervals; square size reflects study weight. The *x*-axis represents specificity values, and the *y*-axis lists the included studies. The pooled specificity was 0.92 (95% CI: 0.75–0.98), based on a bivariate random-effects model. Key takeaway: Specificity remains high on average, suggesting that false-positive rates can be controlled even in prediagnostic settings; however, high heterogeneity limits confidence in generalizability.

### 3.5 Subgroup analyses of diagnostic performance

Given the limited number of studies in several subgroups, these comparisons should be interpreted as descriptive rather than inferential.

To identify which deep learning architectures achieved the best detection performance, we performed subgroup analyses based on two complementary dimensions: (i) model architecture and (ii) integration of clinical prior knowledge. Given the limited number of studies in several subgroups, these comparisons should be interpreted as descriptive rather than inferential. All data were recalculated from the contingency tables (TP, FN, FP, TN) provided in Supplementary Table 2 and verified against the original publications.

#### 3.5.1 Subgroup by model architecture

Studies were classified as pure CNN (2D or 3D CNNs, ResNet, U-Net, nnU-Net), hybrid (CNN-Transformer), or task-enhanced (explicit integration of secondary signs). Table 5 summarises the results.

**Table 5:** Subgroup analysis by model architecture.

Architecture	N	Sensitivity range (median)	Specificity range (median)	Key findings
Pure CNN	11	0.893 (0.860–0.919)	0.944 (0.890–0.972)	Marked drop in cross-racial/external validation (e.g., Liu et al. (21) sensitivity 79%)
Hybrid (CNN + Transformer)	2	0.93–0.95 (0.945)	0.73–0.99 (0.856)	Stable external validation; Cao et al. (37) (PANDA) achieved AUC 0.984 in 10-centre study, sensitivity 92.2% for T1 tumours
Task-enhanced	2	0.92–0.97 (0.925)	0.95–1.00 (0.968)	Degand et al. (38) (PANCANAI): pre-diagnosis sensitivity 68.7% (53.9% for scans >1 year before diagnosis)

CNN-based, hybrid, and task-enhanced models demonstrated varying performance characteristics. Hybrid and task-enhanced models showed improved external validation and early detection performance. Interpretation: These findings support the development of architectures that enhance robustness and generalizability across populations and clinical settings.

For the pure CNN subgroup, a bivariate random-effects model was used to obtain pooled sensitivity and specificity (45). For subgroups with only two studies, only descriptive statistics are reported.

#### 3.5.2 Descriptive comparison by level of clinical prior integration

Based on whether and how clinical priors (e.g., pancreatic duct dilatation, texture features) were incorporated, studies were grouped into strong, weak, or no integration. Table 6 presents the ranges and medians.

**Table 6:** Comparison by level of clinical prior integration.

Integration level	N	Sensitivity range (median)	Specificity range (median)
Strong (explicit duct modelling)	2	0.92–0.97 (0.945)	0.95–1.00 (0.975)
Weak (handcrafted texture or self-attention only)	2	0.93–0.95 (0.94)	0.73–0.99 (0.86)
None (pure data-driven)	11	0.76–0.99 (0.90)	0.70–1.00 (0.95)

Models incorporating explicit clinical priors demonstrated higher specificity and improved early detection performance compared to purely data-driven approaches. Interpretation: Integration of domain-specific clinical knowledge may enhance both model interpretability and diagnostic robustness.

Note on pre-diagnosis sensitivity: Only a few studies reported pre-diagnosis sensitivity. Among strong-integration models, Degand et al. (38) reported 68.7% (overall) and 53.9% (scans >1 year before diagnosis). Among pure data-driven models, only Korfiatis et al. (26) reported 75%. Cross-subgroup comparisons should be made with caution due to limited data.

In summary, the subgroup analysis by model architecture (Table 5) showed that pure CNN models had a pooled sensitivity of 0.893 (95% CI: 0.860–0.919) and pooled specificity of 0.944 (95% CI: 0.890–0.972), but with a marked drop in sensitivity during cross-racial or external validation [e.g., Liu et al. (30) fell to 79%]. Hybrid (CNN-Transformer) models demonstrated stable external validation; notably, Cao et al. (37) (PANDA) achieved an AUC of 0.984 in a 10-centre study with 92.2% sensitivity for T1 tumours. Task-enhanced models appeared to perform well in the pre-diagnosis setting: Degand et al. (38) (PANCANAI) reported an overall pre-diagnosis sensitivity of 68.7% (53.9% for scans acquired >1 year before diagnosis).

The descriptive comparison by level of clinical prior integration (Table 6) further revealed that the strong-integration subgroup (explicit duct modelling) had higher median sensitivity (0.945) and specificity (0.975) than the weak-integration and none-integration subgroups, and also showed a clear advantage in pre-diagnosis sensitivity Degand et al. (38) (68.7%). The weak-integration subgroup (handcrafted texture features or self-attention only) performed intermediately, while the none-integration (pure data-driven) subgroup exhibited the widest variability in specificity (range 0.70–1.00). These findings suggest that explicit encoding of clinical priors (especially main pancreatic duct dilatation) and the adoption of hybrid architectures with a global receptive field may help improve the generalisability and early diagnostic sensitivity of pancreatic cancer detection models.

### 3.6 Results of the exploratory analysis of traditional machine learning models

A total of five traditional machine learning studies were included Chen et al. (53), Chang et al. (49), Chu et al. (50), Javed et al. (51), and Mukherjee et al. (52). The pooled sensitivity was 92.9% (95% CI: 91.0–94.9%) with low heterogeneity (I^2^ = 10.6%); the pooled specificity was 92.7% (95% CI: 85.1–100%, upper bound truncated) with substantial heterogeneity (I^2^ = 94.7%). The detailed data, including 2 × 2 tables, sensitivity, specificity with 95% CIs for each study, as well as the pooled estimates and heterogeneity statistics, are presented in Supplementary Table 3.

3.7 Supplementary analyses: influence, meta-regression, and publication bias.

To further explore sources of heterogeneity and assess the robustness of the pooled estimates, we performed the following supplementary analyses.

Influence analysis (leave-one-out). This leave-one-out influence analysis served as a sensitivity analysis to identify potential outlier or high-risk studies. Iteratively removing each study and refitting the bivariate model showed that the pooled sensitivity and specificity remained stable; the absolute changes ranged from −0.0081 to +0.0042 for sensitivity and from −0.0078 to +0.0066 for specificity. All changes were below the pre-specified influential threshold of 0.02, indicating that no single study disproportionately influenced the summary estimates (Supplementary Table 4). This finding indicates that the pooled estimates are robust to the exclusion of any individual study, and that the potential optimistic bias introduced by selecting the best-performing model per study is unlikely to have materially influenced the overall conclusions.

Meta-regression. Univariable random-effects meta-regression was conducted on logit sensitivity using study design (single-center vs. multicenter) and model architecture (pure CNN/Transformer vs. hybrid vs. task-enhanced) as covariates. A significant association was found for study design: multicenter studies yielded a lower logit sensitivity than single-center studies (coefficient = −0.587, 95% CI: −1.085 to −0.088, *p* = 0.021), explaining 26.1% of the between-study variance (R^2^ = 26.1%). In contrast, model architecture was not significantly associated with sensitivity (coefficient = 0.242, 95% CI: −0.159 to 0.642, *p* = 0.237), likely due to the limited number of studies in the hybrid (*n =* 2) and task-enhanced (*n =* 2) subgroups. Including both covariates together explained 32.7% of the variance (Supplementary Table 5). Residual heterogeneity remained substantial (I^2^ ≈ 82–88%, *p* < 0.001), indicating that other unmeasured study characteristics (e.g., CT protocol, tumour stage distribution) also contribute to the observed heterogeneity.

Publication bias. In addition to Deeks’ funnel plot asymmetry test (*p* = 0.49), we performed Egger’s regression test and Begg’s rank correlation test based on the diagnostic odds ratio. Both tests were not significant (Egger: *t* = 0.59, *p* = 0.567; Begg: *z* = 1.93, *p* = 0.054), suggesting no statistically significant publication bias was detected, although the limited number of studies reduces the power of asymmetry tests.

### 3.7 Subgroup analysis by methodological quality (METRICS)

To examine whether study quality influenced diagnostic estimates, studies were stratified by METRICS quality category (good: *n =* 8; moderate: *n =* 6; low: *n =* 1 excluded). Using a bivariate random-effects model with 5,000 bootstrap resamples, the good-quality subgroup showed a pooled sensitivity of 0.900 and specificity of 0.932; the moderate-quality subgroup showed 0.908 and 0.946, respectively. The differences (Good – Moderate) were −0.0043 (95% CI: −0.0582 to 0.0529, *p* = 0.89) for sensitivity and −0.0242 (95% CI: −0.114 to 0.0364, *p* = 0.56) for specificity, neither statistically significant. Thus, the high overall accuracy was not driven solely by lower-quality studies (Supplementary Table 6). This finding may be partly explained by the nature of the METRICS tool itself, which primarily evaluates reporting completeness and methodological transparency rather than the intrinsic validity or generalizability of model performance. In other words, reporting quality does not necessarily equate to model validity: studies with more comprehensive reporting are not guaranteed to demonstrate superior diagnostic accuracy, and conversely, studies with lower reporting quality may still report high performance.

## 4 Discussion

This systematic review and meta-analysis evaluated the diagnostic performance of deep learning (DL) models for detecting pancreatic ductal adenocarcinoma (PDAC) on CT images. The pooled results demonstrated high diagnostic accuracy in retrospective datasets, with a sensitivity of 0.92, specificity of 0.96, diagnostic odds ratio of 285.00, and a summary area under the receiver operating characteristic curve of 0.97. These findings validate DL as a powerful decision-support tool with significant potential to enhance diagnostic precision for PDAC.

### 4.1 Principal findings and clinical implications

Our results confirm that DL models specifically designed for CT imaging achieve high diagnostic accuracy for PDAC detection. Currently, PDAC diagnosis relies heavily on the subjective interpretation of CT scans by radiologists, a process whose accuracy can be influenced by factors such as experience level (54). Our findings demonstrate that the high sensitivity and specificity of deep learning models could assist radiologists in reducing misdiagnosis and missed diagnoses, particularly for early-stage or atypical lesions. Furthermore, the subgroup analysis of “prediagnostic” images acquired 3 to 36 months prior to clinical diagnosis revealed that the DL models maintained a pooled sensitivity of 0.73 (95% CI: 0.69–0.77) and specificity of 0.92 (95% CI: 0.75–0.98). Although this subgroup was limited in size and exhibited high heterogeneity, necessitating cautious interpretation, this finding is highly suggestive. These findings preliminarily reveal the potential of DL models for early warning of pancreatic cancer, offering new hope for early intervention and improved patient prognosis (55, 56). A note on clinical readiness. Despite the high pooled accuracy, it is important to emphasize that all included studies were retrospective, and only a minority performed external validation on independent datasets. None of the studies assessed deep learning models in prospective clinical workflows or real-time deployment environments. Formal cost-effectiveness analyses and implementation outcome measures (e.g., impact on radiologist workflow, diagnostic confidence, or patient management) were absent. Therefore, while our findings are promising, they should be considered hypothesis-generating and exploratory. The statement “potential integration as clinical decision-support tools” in the Abstract is a forward-looking indication of potential, not an assertion of current readiness. Prospective, multicenter validation studies with embedded AI tools and rigorous implementation science are urgently needed.

### 4.2 Comparison with previous systematic reviews and novelty

Our findings are consistent with those of previous meta-analyses in confirming the high diagnostic accuracy of AI for PDAC detection. However, beyond confirming this known trend, our study provides four specific advances that have not been quantitatively synthesized before, and we have substantially revised this section to articulate them clearly.

First, exclusive focus on pure deep learning (DL) versus mixed AI.

Previous meta-analyses, such as Harandi et al. (17) and Costa et al. (18), included both conventional machine learning (ML) models (random forest, support vector machines, XGBoost) and DL models. Harandi et al. (17) pooled 26 studies (internal validation) and reported a sensitivity of 93% (95% CI: 90–95) and specificity of 95% (95% CI: 92–97), but their subgroup analysis showed that random forest achieved the highest sensitivity (98%) whereas CNN had the highest specificity (96%) (17). Costa et al. (18) included nine studies (mostly DL) and reported a sensitivity of 87.9% (95% CI: 82.9–91.6) and specificity of 92.2% (95% CI: 86.8–95.5). By focusing exclusively on deep learning and excluding traditional ML algorithms, our study provides a purer estimate of the current state-of-the-art AI technology, uncontaminated by models that are no longer representative of modern clinical image analysis.

Second, first head-to-head comparison across deep learning architectures.

Neither Harandi et al. (17) nor Costa et al. (18) performed subgroup analyses based on DL architectural differences (e.g., pure CNN vs. hybrid CNN-Transformer vs. task-enhanced). Our subgroup analysis (Table 5) and meta-regression reveal, for the first time, that these architectures have distinct performance profiles: pure CNNs show high accuracy on internal data but a marked drop during external validation; hybrid CNN-Transformer models maintain stable generalizability across centers (e.g., PANDA achieved an AUC of 0.984 in a 10-centre study (37)); and task-enhanced models that explicitly encode clinical priors (e.g., main pancreatic duct dilatation) achieve the highest pre-diagnosis sensitivity (68.7% overall, 53.9% for scans >1 year before diagnosis) (38). This mechanistic insight—linking architectural properties (local vs. global receptive fields, explicit clinical prior encoding) to generalizability and early detection—goes beyond confirming known performance trends and offers actionable guidance for model selection in future clinical implementations.

Third, first pooled estimates of prediagnostic (early) detection performance.

Harandi et al. (17) explicitly excluded studies that predicted PDAC before diagnosis, focusing only on diagnostic scans. Costa et al. (18) included two pre-diagnosis studies but did not perform a dedicated subgroup analysis to pool their estimates. Our study provides the first quantitative synthesis of DL performance on scans acquired 3–36 months before clinical diagnosis (sensitivity 73, 95% CI: 69–77%; specificity 92, 95% CI: 75–98%; Section 3.4), offering a baseline for future early warning systems.

Fourth, comprehensive quality assessment with novel tools (METRICS).

Both previous meta-analyses used QUADAS-2 and PROBAST or RQS, but neither applied the recently developed METRICS tool, which provides expert-weighted scores across 30 items. We used METRICS alongside QUADAS-2, RQS, and CLAIM to identify specific methodological gaps (e.g., 80% of studies failed to report feature extraction parameters; none removed redundant features; open science practices were severely lacking). These findings have not been systematically reported before and highlight critical areas for improvement in future research (Section 3.3).

Taken together, while our pooled sensitivity and specificity are similar in magnitude to previous reports, the granularity of architectural comparison, the first quantification of prediagnostic performance, and the detailed METRICS-based quality assessment represent significant methodological and clinical advances. We have also tempered our conclusions regarding clinical readiness (revised Abstract and Conclusion) to more accurately reflect the retrospective nature of the included studies.

### 4.3 Comparison with traditional machine learning approaches

The exploratory meta-analysis (see Supplementary Table 3) of five traditional machine learning studies yielded a pooled sensitivity of 92.9% and a pooled specificity of 92.7%. Compared with the pooled results of deep learning models in our primary analysis (sensitivity 92.0%, specificity 96.0%), the two approaches had similar sensitivity, whereas deep learning showed higher specificity (96.0% vs. 92.7%) and a higher diagnostic odds ratio (285 vs. 150). Furthermore, indirect comparisons with the traditional machine learning subgroup data from published meta-analyses (17, 18) showed consistent trends. These findings suggest that deep learning models maintain high sensitivity while offering superior specificity, which may be advantageous in reducing false-positive findings. However, this comparison is indirect, and the exploratory analysis of traditional machine learning models was limited by the small number of included studies and high heterogeneity in specificity. Prospective head-to-head studies are needed to validate these observations.

### 4.4 In-depth assessment of methodological quality and reporting transparency

Our multitool assessment strategy provided a multifaceted perspective on the quality of the included studies. The QUADAS-2 assessment indicated that the retrospective design introduced a risk of selection bias, which is currently prevalent in this field (57). More instructive findings emerged from the evaluation of radiomics and AI-specific reporting standards. The RQS assessment showed an average score of 45.60% for the included studies, which was higher than the systematic average (18.87%) reported by the EuSoMII Auditing Group, potentially attributable to the low reproducibility of the RQS itself (58). Conversely, the average score from the newly proposed METRICS tool (62.21%) suggests that RQS might underestimate study quality, whereas METRICS might overestimate it. This discrepancy between tools indicates that future research should refer to the latest standards, such as the CLEAR guidelines endorsed by the ESR and EuSoMII (59), to further enhance reporting quality and methodological rigor. The CLAIM checklist evaluation further revealed that although most studies covered key aspects of AI research, there is substantial room for improvement in the transparency of reporting data preprocessing steps, the rationale for hyperparameter selection, and the sharing of code and models (60). This transparency is crucial for model reproducibility and clinical translation.

Notably, a subgroup analysis stratified by METRICS quality category (Section 3.8) revealed no significant differences in sensitivity or specificity between good- and moderate-quality studies, confirming that the high diagnostic accuracy is not attributable to lower-quality studies.

### 4.5 Interpretation of subgroup findings: why certain architectures perform better

It should be noted that the subgroup findings are descriptive in nature and should be interpreted with caution due to the limited number of studies and the absence of direct head-to-head comparisons. Our subgroup analyses reveal consistent patterns that can be explained by the mathematical properties of the models.

Pure CNNs rely on a local receptive field. They perform well on homogeneous internal data but fail to capture long-range secondary signs (e.g., upstream duct dilatation, parenchymal atrophy). This explains the marked sensitivity drop during cross-racial or external validation [e.g. Liu et al. (21): 79% on a US test set]. Hybrid CNN-Transformer models use self-attention to obtain a global receptive field, enabling simultaneous analysis of the tumour and distant indirect signs. This may contribute to more stable performance in external validation across different scanners and populations. Cao et al. (37) (PANDA) achieved an AUC of 0.984 in a 10-centre study, with 92.2% sensitivity for T1 tumours. Task-enhanced models explicitly encode clinical rules. Degand et al. (38) (PANCANAI) separate lesion detection and main pancreatic duct dilatation; a positive result from either branch triggers an alert, exploiting the knowledge that ductal dilatation can precede visible tumour by months. This yields the highest reported pre-diagnosis sensitivity (68.7% overall; 53.9% for scans >1 year before diagnosis) with very high specificity (95–100%). Similarly, Ramaekers et al. (29) explicitly fed ductal segmentation maps into the network, achieving near-perfect specificity. From the perspective of clinical prior integration, strong integration (explicit duct modelling) consistently improves specificity and pre-diagnosis sensitivity compared with pure data-driven approaches. Weak integration (handcrafted texture features or implicit self-attention) offers intermediate benefits. In summary, global receptive fields (via self-attention) enhance generalisability by capturing long-range indirect signs, while explicit encoding of clinical priors (e.g., duct dilatation) may improve early detection. Pure CNNs, though highly accurate on curated internal data, are limited by local receptive fields and show performance degradation in cross-centre and early-screening scenarios.

### 4.6 In-depth analysis of heterogeneity sources

We detected significant heterogeneity among the included studies (I^2^ for sensitivity = 87.4%; I^2^ for specificity = 95.9%). To trace its origins, we performed detailed subgroup analyses, which identified study design, AI model architecture, and CT scanning parameters as major contributing factors (61). Specifically, multicenter studies demonstrated lower heterogeneity than single-center studies did (as indicated by reduced I^2^ values in subgroup analysis). At the model level, subgroup analyses focusing on specific architectures also revealed reduced heterogeneity. These findings provide clear guidance for future research: adopting prospective, multicenter designs, along with detailed reporting and standardization of image acquisition protocols and model selection, can significantly improve the consistency, comparability, and reliability of future research outcomes. Our decomposition of heterogeneity using the Zhou–Dendukuri bivariate I^2^ (37.1%) indicates that the conventional unadjusted I^2^ values (87–96%) overestimate the true between-study variation. The large reduction after adjusting for sample size (Holling adjusted I^2^ = 1.8–2.9%) confirms that much of the apparent heterogeneity is artefactual, arising from differences in sample size and threshold effects rather than fundamental inconsistency in model performance. Meta-regression further identified study design (multicenter vs. single-center) as a significant moderator of sensitivity (*p* = 0.021), explaining 26% of the variance. Although study design was identified as a statistically significant source of heterogeneity, it explained only 26.1% of the between-study variance, and substantial residual heterogeneity remained. Therefore, these findings should be interpreted cautiously. Rather than providing definitive guidance, they suggest that prospective multicenter studies with standardized image acquisition protocols may improve the robustness and generalizability of future research.

### 4.7 Beyond imaging: toward multi-modal and responsible AI

While the present findings highlight the strong diagnostic performance of deep learning models applied to CT imaging, it is important to contextualize these results within the broader complexity of pancreatic cancer biology and real-world clinical practice.

First, imaging-based models capture only a partial representation of disease processes. As emphasized by Tan et al. (62), health and disease emergence are shaped by dynamic, real-time interactions among genetic, environmental, and lifestyle factors over time. In this context, CT-derived features primarily reflect downstream structural and morphological manifestations, rather than upstream biological or behavioral determinants. Consequently, the high performance observed in imaging-based models should not be interpreted as a comprehensive representation of disease detection capability. Future approaches should move toward multi-modal frameworks that integrate imaging with clinical features (e.g., new-onset diabetes, weight loss, CA19-9), and, where available, longitudinal electronic health record data to enable more robust and personalized detection. Such integration may facilitate a shift from isolated diagnostic tools toward a broader predictive ecosystem for early cancer detection. This transition will likely require tighter integration between AI systems and multidisciplinary clinical infrastructures, including tumor board-centered decision-making processes.

Second, important challenges related to fairness, bias, and interpretability remain insufficiently addressed in the current literature. As highlighted by Tan and Benos (63), addressing intersectionality, explainability, and ethical considerations is essential for the responsible deployment of AI systems in clinical medicine. All included studies were retrospective and conducted predominantly in limited geographic regions, with no reporting of subgroup-specific performance by race, ethnicity, socioeconomic status, or comorbidity burden. As a result, pooled estimates may mask substantial disparities across patient populations. Dataset imbalances, spectrum bias, and variations in imaging protocols further limit generalizability to real-world clinical settings.

In addition, interpretability remains a critical barrier to clinical adoption. While some models provide saliency maps or attention visualizations, most studies lack rigorous explainable AI approaches, limiting transparency and clinician trust (64). Without clear insight into model decision-making processes, hidden biases may remain undetected, further compounding risks in clinical deployment.

Taken together, these considerations suggest that future progress in AI-assisted PDAC detection should move beyond optimizing diagnostic accuracy alone, toward developing clinically integrated, interpretable, and equitable systems that better reflect the complexity of real-world disease. Importantly, high pooled diagnostic accuracy does not necessarily translate into equitable performance across diverse patient populations, underscoring the need for responsible and inclusive AI development.

More broadly, the future role of artificial intelligence in pancreatic cancer care should not be viewed as isolated algorithmic prediction, but rather as part of a transdisciplinary clinical decision-support ecosystem. As highlighted by Tan et al. (64), effective integration of AI into oncology requires close collaboration among radiologists, oncologists, surgeons, pathologists, bioinformaticians, and data scientists within multidisciplinary clinical workflows such as tumor boards. In this context, data scientists function not merely as technical developers, but as integrators of heterogeneous data streams—including imaging, clinical, genomic, and longitudinal health information—to support more context-aware and personalized decision-making. In addition, causal reasoning frameworks may help improve the interpretability, robustness, and clinical reliability of AI-assisted decision support systems. Such a systems-level approach may be particularly important for pancreatic cancer, where diagnosis and treatment planning often depend on subtle interactions among imaging findings, biological markers, patient comorbidities, and temporal disease evolution. Therefore, future progress should emphasize clinically embedded, interpretable, and workflow-aware AI systems that augment multidisciplinary collaboration rather than operate as standalone diagnostic tools.

### 4.8 Limitations and future perspectives

This study has several limitations. First, all included studies were retrospective in design, carrying an inherent risk of selection bias (65). Moreover, the evidence is exclusively retrospective and lacks real-world validation. All 15 included studies were retrospective in design, which carries inherent risks of selection bias, spectrum bias (i.e., the case mix may not reflect real-world patient populations), and dataset bias (e.g., enrichment with advanced tumors, specific CT protocols, or particular ethnic groups). External validation was performed in only a subset of studies (*n =* 7) and was inconsistent in terms of population characteristics and imaging equipment. No study evaluated deep learning models in a prospective clinical workflow or real-time deployment setting. Consequently, the generalizability of the pooled estimates to routine clinical practice remains unproven. Formal cost-effectiveness analyses and assessments of implementation barriers (e.g., integration with electronic health records, radiologist acceptance) were also absent. These limitations substantially temper the readiness of deep learning models for clinical integration and highlight the urgent need for prospective, multicenter implementation studies.

Second, only a limited number of studies have investigated performance on “prediagnostic” images, restricting our ability to accurately assess the role of DL in early screening. Furthermore, only one study was based on noncontrast CT, which is a widely used modality in clinical practice because of its convenience and cost-effectiveness. Future studies are needed to validate the performance of DL on noncontrast CT.

Third, we did not perform meta-regression or quality-weighted pooling based on METRICS scores because the composite score lacks a theoretical linear relationship with diagnostic performance, and no validated weighting framework exists. However, our subgroup analysis (Section 3.8) showed no significant performance differences between good- and moderate-quality studies, supporting robustness.

Another important limitation relates to the handling of multiple models within individual studies. To preserve statistical independence, only one representative model per study was included in the primary meta-analysis, following a predefined hierarchical selection strategy based on AUC and diagnostic accuracy. Although this approach is commonly used in diagnostic meta-analyses addressing effect-size multiplicity, it may preferentially retain the most favorable results and therefore introduce optimistic bias into the pooled estimates. Because most included studies did not report complete 2 × 2 contingency data for all evaluated models, a formal multilevel or multi-model meta-analysis could not be reliably performed. Consequently, the pooled diagnostic performance reported in this study may reflect relatively optimistic estimates of real-world clinical performance of deep learning systems. Future meta-analyses using hierarchical modeling frameworks and comprehensive model-level reporting are needed to address this issue more rigorously.

On the basis of current research, future directions can be summarized as follows: First, prospective multicenter validation should be actively promoted to ensure model generalizability and clinical utility. Second, efforts to enhance standardization and reproducibility are needed, including standardized CT scan parameters and contrast protocols, improved segmentation methods, and encouraged sharing of data and code. Furthermore, research should move beyond diagnostic accuracy to evaluate differences across scanners and vendors and conduct cost-effectiveness analyses to demonstrate the real-world value of deep learning-based decision support systems. Finally, it is essential to explore seamless integration of AI tools into existing clinical workflows and assess their practical impact on radiologists’ workflow efficiency and diagnostic confidence.

## 5 Conclusion

In summary, this systematic review and meta-analysis demonstrates that deep learning models achieve high diagnostic accuracy for detecting PDAC on CT scans in retrospective datasets. Nevertheless, the evidence remains exploratory due to several critical limitations: all included studies were retrospective, external validation was inconsistent, no prospective or real-world deployment data were available, and cost-effectiveness was not evaluated. Potential dataset and spectrum biases may also affect generalizability. Therefore, while our findings suggest potential for DL as a decision-support tool, they do not support current clinical integration. Future prospective, multicenter validation studies with standardized imaging protocols, real-world workflow integration, and formal cost-effectiveness analyses are essential before any clinical implementation can be considered. In addition, because only the best-performing model from each study was included, the pooled estimates may reflect relatively optimistic estimates of diagnostic performance rather than average real-world clinical effectiveness.
