# Supplementary material for: Deep learning models for pancreatic cancer detection on CT: a meta-analysis
Source: Front Med (Lausanne). 2026 Sep 14;13:1826557. doi: 10.3389/fmed.2026.1826557 (PMC13616657; doi:10.3389/fmed.2026.1826557)
Supplement: Supplementary file 1 [file Table_1.DOCX]

\# ============================================================

\# Supplementary Code 1

\# R scripts for supplementary analyses: bivariate model,

\# meta-regression, leave-one-out, publication bias tests,

\# and METRICS quality subgroup analysis.

\#

\# Manuscript: "Deep Learning Models for Pancreatic Cancer Detection on CT: A Meta-Analysis"

\#

\# This script is fully self-contained (data embedded).

\# Required packages: mada, meta, metafor

\# ============================================================

\# ------------------------------

\# 0. Install and load required packages

\# ------------------------------

if (!require("mada")) install.packages("mada")

if (!require("meta")) install.packages("meta")

if (!require("metafor")) install.packages("metafor")

library(mada)

library(meta)

library(metafor)

\# ------------------------------

\# 1. Data: 15 studies with 2x2 tables

\# ------------------------------

my_data <- data.frame(

study = c("Korfiatis2023", "Liu2020", "Chen2023", "Cao2023",

"QU2024", "Ma2020", "Si2021", "Park2023", "Ramaekers2024",

"Viriyasaranon2023", "Mandal2024", "Liao2025", "Thanya2025",

"Degand2025", "Nadeem2025"),

TP = c(360, 395, 698, 2722, 409, 203, 148, 81, 96, 230, 162, 122, 163, 1075, 152),

FN = c(49, 62, 80, 186, 67, 19, 22, 12, 3, 51, 17, 6, 4, 145, 1),

FP = c(41, 3, 64, 31, 31, 3, 25, 177, 0, 0, 16, 26, 2, 109, 0),

TN = c(788, 231, 887, 2721, 260, 187, 57, 922, 98, 80, 163, 68, 72, 1600, 130)

)

\# Add covariates for meta-regression

my_data$design <- c(1,0,0,0,1,0,1,1,0,1,1,1,0,1,0) # 1=multicenter, 0=single-center

my_data$model_type <- factor(c(1,1,1,2,1,1,1,1,3,1,1,2,1,3,1),

labels = c("pure","hybrid","taskEnhanced"))

\# ------------------------------

\# 2. Bivariate random-effects model (Reitsma)

\# ------------------------------

reitsma_fit <- reitsma(data = my_data[, c("TP","FN","FP","TN")])

summary(reitsma_fit)

\# Extract pooled sensitivity and specificity (point estimates and 95% CI)

coef_sum <- summary(reitsma_fit)$coefficients

logit_sens <- coef_sum[1,1]; se_logit_sens <- coef_sum[1,2]

logit_fpr <- coef_sum[2,1]; se_logit_fpr <- coef_sum[2,2]

sens <- exp(logit_sens) / (1 + exp(logit_sens))

sens_low <- exp(logit_sens - 1.96*se_logit_sens) / (1 + exp(logit_sens - 1.96*se_logit_sens))

sens_up <- exp(logit_sens + 1.96*se_logit_sens) / (1 + exp(logit_sens + 1.96*se_logit_sens))

spec <- 1 - (exp(logit_fpr) / (1 + exp(logit_fpr)))

spec_low <- 1 - (exp(logit_fpr + 1.96*se_logit_fpr) / (1 + exp(logit_fpr + 1.96*se_logit_fpr)))

spec_up <- 1 - (exp(logit_fpr - 1.96*se_logit_fpr) / (1 + exp(logit_fpr - 1.96*se_logit_fpr)))

cat("\n========== Pooled results ==========\n")

cat(sprintf("Sensitivity: %.3f (95%% CI: %.3f-%.3f)\n", sens, sens_low, sens_up))

cat(sprintf("Specificity: %.3f (95%% CI: %.3f-%.3f)\n", spec, spec_low, spec_up))

\# Diagnostic odds ratio (DOR)

logDOR <- logit_sens - logit_fpr

se_logDOR <- sqrt(se_logit_sens^2 + se_logit_fpr^2)

dor <- exp(logDOR)

dor_low <- exp(logDOR - 1.96*se_logDOR)

dor_up <- exp(logDOR + 1.96*se_logDOR)

cat(sprintf("Diagnostic odds ratio (DOR): %.0f (95%% CI: %.0f-%.0f)\n", dor, dor_low, dor_up))

\# ------------------------------

\# 3. SROC curve (Figure 4)

\# ------------------------------

sroc_obj <- sroc(reitsma_fit)

png("Figure_4_SROC.png", width = 8, height = 6, units = "in", res = 300)

plot(sroc_obj, col = "blue", lwd = 2,

xlab = "1 - Specificity", ylab = "Sensitivity",

main = "Summary ROC Curve")

dev.off()

cat("\nSROC curve saved as Figure_4_SROC.png\n")

\# ------------------------------

\# 4. Leave-one-out influence analysis

\# ------------------------------

extract_sens_spec <- function(fit) {

logit_sens <- fit$coefficients[1]

logit_fpr <- fit$coefficients[2]

sens <- exp(logit_sens) / (1 + exp(logit_sens))

spec <- 1 - (exp(logit_fpr) / (1 + exp(logit_fpr)))

return(c(sens = sens, spec = spec))

}

n <- nrow(my_data)

sens_loo <- numeric(n)

spec_loo <- numeric(n)

for (i in 1:n) {

loo_data <- my_data[-i, c("TP","FN","FP","TN")]

loo_fit <- reitsma(loo_data)

tmp <- extract_sens_spec(loo_fit)

sens_loo[i] <- tmp["sens"]

spec_loo[i] <- tmp["spec"]

}

diff_sens <- sens_loo - sens

diff_spec <- spec_loo - spec

cat("\n========== Leave-one-out results ==========\n")

cat(sprintf("Max absolute change in sensitivity: %.4f\n", max(abs(diff_sens))))

cat(sprintf("Max absolute change in specificity: %.4f\n", max(abs(diff_spec))))

\# Create a table for Supplementary Table 4 (leave-one-out)

loo_table <- data.frame(

Study_removed = my_data$study,

Delta_sensitivity = round(diff_sens, 4),

Delta_specificity = round(diff_spec, 4)

)

write.csv(loo_table, "Supplementary_Table4_leave_one_out.csv", row.names = FALSE)

cat("Supplementary Table 4 (leave-one-out) saved as Supplementary_Table4_leave_one_out.csv\n")

\# ------------------------------

\# 5. Meta-regression (on logit sensitivity)

\# ------------------------------

my_data$sens_raw <- my_data$TP / (my_data$TP + my_data$FN)

my_data$logit_sens <- log(my_data$sens_raw / (1 - my_data$sens_raw))

my_data$se_logit_sens <- sqrt(1/my_data$TP + 1/my_data$FN)

\# Covariate: study design

rma_design <- rma(yi = logit_sens, sei = se_logit_sens, mods = ~ design, data = my_data, method = "REML")

cat("\n========== Meta-regression (study design) ==========\n")

print(summary(rma_design))

\# Covariate: model type (as numeric)

my_data$model_num <- as.numeric(my_data$model_type)

rma_model <- rma(yi = logit_sens, sei = se_logit_sens, mods = ~ model_num, data = my_data, method = "REML")

cat("\n========== Meta-regression (model type) ==========\n")

print(summary(rma_model))

\# Save meta-regression results for Supplementary Table 5

meta_reg_results <- data.frame(

Analysis = c("Meta-regression (design)", "Meta-regression (model type)"),

Covariate = c("multicenter vs single-center", "model type (pure→hybrid→taskEnhanced)"),

Estimate = c(coef(summary(rma_design))[2,1], coef(summary(rma_model))[2,1]),

CI_lower = c(coef(summary(rma_design))[2,1] - 1.96*coef(summary(rma_design))[2,2],

coef(summary(rma_model))[2,1] - 1.96*coef(summary(rma_model))[2,2]),

CI_upper = c(coef(summary(rma_design))[2,1] + 1.96*coef(summary(rma_design))[2,2],

coef(summary(rma_model))[2,1] + 1.96*coef(summary(rma_model))[2,2]),

p_value = c(coef(summary(rma_design))[2,4], coef(summary(rma_model))[2,4])

)

write.csv(meta_reg_results, "Supplementary_Table5_meta_regression.csv", row.names = FALSE)

cat("Supplementary Table 5 (meta-regression) saved as Supplementary_Table5_meta_regression.csv\n")

\# ------------------------------

\# 6. Publication bias tests (Egger, Begg, Deeks)

\# ------------------------------

\# Compute DOR and its standard error (with continuity correction for zero cells)

my_data$DOR <- (my_data$TP/my_data$FN) / (my_data$FP/my_data$TN)

my_data$logDOR <- log(my_data$DOR)

my_data$se_logDOR <- sqrt(1/my_data$TP + 1/my_data$FN + 1/my_data$FP + 1/my_data$TN)

for (i in 1:n) {

if (my_data$FP[i] == 0 | my_data$FN[i] == 0) {

my_data$logDOR[i] <- log((my_data$TP[i]+0.5)/(my_data$FN[i]+0.5) / ((my_data$FP[i]+0.5)/(my_data$TN[i]+0.5)))

my_data$se_logDOR[i] <- sqrt(1/(my_data$TP[i]+0.5) + 1/(my_data$FN[i]+0.5) + 1/(my_data$FP[i]+0.5) + 1/(my_data$TN[i]+0.5))

}

}

meta_dor <- metagen(TE = logDOR, seTE = se_logDOR, data = my_data,

studlab = study, sm = "OR", common = FALSE, random = TRUE)

\# Egger test

egger <- metabias(meta_dor, method = "Egger", k.min = 5)

cat("\n========== Egger test ==========\n")

print(egger)

\# Begg test

begg <- metabias(meta_dor, method = "Begg", k.min = 5)

cat("\n========== Begg test ==========\n")

print(begg)

\# Deeks funnel plot (Supplementary Figure 1)

png("Figure_S1_Deeks_funnel.png", width = 6, height = 6, units = "in", res = 300)

funnel(meta_dor, xlab = "Log Odds Ratio", main = "Funnel Plot for DOR")

dev.off()

cat("Deeks funnel plot saved as Figure_S1_Deeks_funnel.png\n")

\# Save publication bias results for Supplementary Table 5 (Egger, Begg)

pub_bias_results <- data.frame(

Test = c("Egger's test", "Begg's test"),

Statistic = c(egger$statistic, begg$statistic),

p_value = c(egger$pval, begg$pval)

)

write.csv(pub_bias_results, "Supplementary_Table5_publication_bias.csv", row.names = FALSE)

\# ------------------------------

\# 7. METRICS quality subgroup analysis (good vs moderate)

\# ------------------------------

\# According to Table 3 of the manuscript:

good_studies <- c("Liu2020","Cao2023","Ma2020","Si2021","Park2023","Mandal2024","Liao2025","Degand2025")

moderate_studies <- c("Korfiatis2023","Chen2023","QU2024","Ramaekers2024","Viriyasaranon2023","Thanya2025")

data_good <- my_data[my_data$study %in% good_studies, c("TP","FN","FP","TN")]

data_moderate <- my_data[my_data$study %in% moderate_studies, c("TP","FN","FP","TN")]

fit_good <- reitsma(data_good)

fit_mod <- reitsma(data_moderate)

sens_good <- extract_sens_spec(fit_good)[1]

spec_good <- extract_sens_spec(fit_good)[2]

sens_mod <- extract_sens_spec(fit_mod)[1]

spec_mod <- extract_sens_spec(fit_mod)[2]

cat("\n========== METRICS quality subgroup analysis ==========\n")

cat(sprintf("Good quality (n=8): Sensitivity = %.3f, Specificity = %.3f\n", sens_good, spec_good))

cat(sprintf("Moderate quality (n=6): Sensitivity = %.3f, Specificity = %.3f\n", sens_mod, spec_mod))

\# Bootstrap difference (optional, simplified p-value)

set.seed(123)

n_boot <- 1000

boot_diff_sens <- numeric(n_boot)

boot_diff_spec <- numeric(n_boot)

for (i in 1:n_boot) {

\# Bootstrap good group

idx_good <- sample(1:nrow(data_good), replace = TRUE)

boot_good <- data_good[idx_good, ]

fit_good_boot <- tryCatch(reitsma(boot_good), error = function(e) NULL)

if (!is.null(fit_good_boot)) {

s_good <- extract_sens_spec(fit_good_boot)[1]

p_good <- extract_sens_spec(fit_good_boot)[2]

} else { s_good <- NA; p_good <- NA }

\# Bootstrap moderate group

idx_mod <- sample(1:nrow(data_moderate), replace = TRUE)

boot_mod <- data_moderate[idx_mod, ]

fit_mod_boot <- tryCatch(reitsma(boot_mod), error = function(e) NULL)

if (!is.null(fit_mod_boot)) {

s_mod <- extract_sens_spec(fit_mod_boot)[1]

p_mod <- extract_sens_spec(fit_mod_boot)[2]

} else { s_mod <- NA; p_mod <- NA }

boot_diff_sens[i] <- s_good - s_mod

boot_diff_spec[i] <- p_good - p_mod

}

boot_diff_sens <- boot_diff_sens[!is.na(boot_diff_sens)]

boot_diff_spec <- boot_diff_spec[!is.na(boot_diff_spec)]

p_sens <- 2 * min(mean(boot_diff_sens > 0, na.rm=TRUE), mean(boot_diff_sens < 0, na.rm=TRUE))

p_spec <- 2 * min(mean(boot_diff_spec > 0, na.rm=TRUE), mean(boot_diff_spec < 0, na.rm=TRUE))

cat(sprintf("Sensitivity difference p = %.4f\n", p_sens))

cat(sprintf("Specificity difference p = %.4f\n", p_spec))

\# Save METRICS subgroup results for Supplementary Table 6

metrics_subgroup <- data.frame(

Quality = c("Good", "Moderate"),

N = c(8, 6),

Sensitivity = c(sens_good, sens_mod),

Specificity = c(spec_good, spec_mod)

)

write.csv(metrics_subgroup, "Supplementary_Table6_METRICS_subgroup.csv", row.names = FALSE)

cat("Supplementary Table 6 (METRICS subgroup) saved as Supplementary_Table6_METRICS_subgroup.csv\n")

\# ------------------------------

\# 8. Forest plots for sensitivity and specificity (Figures 3a and 3b)

\# ------------------------------

\# Sensitivity forest plot

my_data$event_sens <- my_data$TP

my_data$n_sens <- my_data$TP + my_data$FN

meta_sens <- metaprop(event = event_sens, n = n_sens, data = my_data,

studlab = study, sm = "PLOGIT",

common = FALSE, random = TRUE,

method.tau = "DL")

png("Figure_3a_forest_sensitivity.png", width = 8, height = 10, units = "in", res = 300)

forest(meta_sens,

xlim = c(0, 1),

xlab = "Sensitivity",

leftcols = c("studlab", "event_sens", "n_sens"),

leftlabs = c("Study", "TP", "Total"),

rightcols = c("effect", "ci", "w.random"),

rightlabs = c("Sensitivity", "95% CI", "Weight"))

dev.off()

cat("Sensitivity forest plot saved as Figure_3a_forest_sensitivity.png\n")

\# Specificity forest plot

my_data$event_spec <- my_data$TN

my_data$n_spec <- my_data$TN + my_data$FP

meta_spec <- metaprop(event = event_spec, n = n_spec, data = my_data,

studlab = study, sm = "PLOGIT",

common = FALSE, random = TRUE,

method.tau = "DL")

png("Figure_3b_forest_specificity.png", width = 8, height = 10, units = "in", res = 300)

forest(meta_spec,

xlim = c(0, 1),

xlab = "Specificity",

leftcols = c("studlab", "event_spec", "n_spec"),

leftlabs = c("Study", "TN", "Total"),

rightcols = c("effect", "ci", "w.random"),

rightlabs = c("Specificity", "95% CI", "Weight"))

dev.off()

cat("Specificity forest plot saved as Figure_3b_forest_specificity.png\n")

\# ------------------------------

\# End of script

\# ------------------------------

cat("\n========== All analyses completed ==========\n")
