# Supplementary material for: Deep learning models for pancreatic cancer detection on CT: a meta-analysis
Source: Front Med (Lausanne). 2026 Sep 14;13:1826557. doi: 10.3389/fmed.2026.1826557 (PMC13616657; doi:10.3389/fmed.2026.1826557)
Supplement: Supplementary file 2 [file Table_2.DOCX]

Supplementary Table 1.Detailed search strategy for each database.

search terms：

**((Neoplasm, Pancreatic) OR(Pancreatic Neoplasm) OR(Neoplasms, Pancreatic) OR(Pancreas Neoplasms) OR(Neoplasm, Pancreas) OR(Neoplasms, Pancreas) OR(Pancreas Neoplasm) OR(Cancer of Pancreas) OR(Pancreas Cancers) OR(Cancer of the Pancreas) OR(Pancreas Cancer) OR(Cancer, Pancreas) OR(Cancers, Pancreas) OR(Pancreatic Cancer) OR(Cancer, Pancreatic) OR(Cancers, Pancreatic) OR(Pancreatic Cancers) OR(Pancreatic Carcinoma) OR(Carcinoma, Pancreatic) OR(Carcinomas, Pancreatic) OR(Pancreatic Carcinomas) OR(Pancreatic Acinar Carcinoma) OR(Acinar Carcinoma, Pancreatic) OR(Acinar Carcinomas, Pancreatic) OR(Carcinoma, Pancreatic Acinar) OR(Carcinomas, Pancreatic Acinar) OR(Pancreatic Acinar Carcinomas) OR(Pancreatic Neoplasms)) AND ((Intelligence, Artificial) OR(Computer Reasoning) OR(Reasoning, Computer) OR(AI (Artificial Intelligence)) OR(Machine Intelligence) OR(Intelligence, Machine) OR(Computational Intelligence) OR(Intelligence, Computational) OR(Computer Vision Systems) OR(Computer Vision System) OR(System, Computer Vision) OR(Systems, Computer Vision) OR(Vision System, Computer) OR(Vision Systems, Computer) OR(Knowledge Acquisition (Computer)) OR(Acquisition, Knowledge (Computer)) OR(Knowledge Representation (Computer)) OR(Knowledge Representations (Computer)) OR(Representation, Knowledge (Computer)) OR(Artificial Intelligence) OR(Learning, Deep) OR(Hierarchical Learning) OR(Learning, Hierarchical) OR(Deep Learning) OR(Convolutional Neural Networks) OR(CNN)) AND ((Computed Tomography) OR (CT))**
