# Supplementary material for: Deep learning models for pancreatic cancer detection on CT: a meta-analysis
Source: Front Med (Lausanne). 2026 Sep 14;13:1826557. doi: 10.3389/fmed.2026.1826557 (PMC13616657; doi:10.3389/fmed.2026.1826557)
Supplement: Supplementary file 3 [file Table_3.DOCX]

| Supplementary Table 2.Individual study 2×2 contingency data (TP, FN, FP, TN) and pooled diagnostic performance of deep learning models. | | | | |
| --- | --- | --- | --- | --- |
|  |  |  |  |  |
|  |  |  |  |  |
| author,year | TP | FP | FN | TN |
| [Korfiatis,2023](https://pubmed.ncbi.nlm.nih.gov/?sort=jour&term=Korfiatis+P&cauthor_id=37657758) | 360 | 41 | 49 | 788 |
| [Liu,2020](https://pubmed.ncbi.nlm.nih.gov/?sort=jour&term=Liu+KL&cauthor_id=33328124) | 395 | 3 | 62 | 231 |
| [Chen,2023](https://pubmed.ncbi.nlm.nih.gov/?sort=jour&term=Chen+PT&cauthor_id=36098642) | 698 | 64 | 80 | 887 |
| [Cao,2023](https://pubmed.ncbi.nlm.nih.gov/?sort=jour&term=Cao+K&cauthor_id=37985692) | 2722 | 31 | 186 | 2721 |
| QU,2024 | 409 | 31 | 67 | 260 |
| Ma,2020 | 203 | 3 | 19 | 187 |
| Si,2021 | 148 | 25 | 22 | 57 |
| Park,2023 | 81 | 177 | 12 | 922 |
| Ramaekers,2024 | 96 | 0 | 3 | 98 |
| Viriyasaranon,2023 | 230 | 0 | 51 | 80 |
| Mandal,2024 | 162 | 16 | 17 | 163 |
| Liao,2025 | 122 | 26 | 6 | 68 |
| Thanya, 2025 | 163 | 2 | 4 | 72 |
| Degand,2025 | 1075 | 109 | 145 | 1600 |
| Nadeem，2025 | 152 | 0 | 1 | 130 |
| Parameter | | Estimate |  | 95% CI |
| Sensitivity | | 0.92 |  | [0.88, 0.94] |
| Specificity | | 0.96 |  | [0.92,0.98] |
| Positive Likelihood Ratio | | 25.1 |  | [10.6,59.0] |
| Negative Likelihood Ratio | | 0.09 |  | [0.06,0.12] |
| Diagnostic Odds Ratio | | 285 |  | [ 97,839] |
| TP = true positive, FP = false positive, FN = false negative, TN = true negative. Pooled estimates were obtained using a bivariate random‑effects model. Values in brackets are 95% confidence intervals. | | | | |
|  |  |  |  |  |
|  |  |  |  |  |
|  |  |  |  |  |
