# Supplementary material for: Deep learning models for pancreatic cancer detection on CT: a meta-analysis
Source: Front Med (Lausanne). 2026 Sep 14;13:1826557. doi: 10.3389/fmed.2026.1826557 (PMC13616657; doi:10.3389/fmed.2026.1826557)
Supplement: Supplementary file 4 [file Table_4.DOCX]

| Supplementary Table 3.Diagnostic performance of the five traditional machine learning studies for PDAC detection on CT. | | | | | | |
| --- | --- | --- | --- | --- | --- | --- |
| Study | TP | FP | FN | TN | Sensitivity (%) (95% CI) | Specificity (%) (95% CI) |
| Chen,2021 | 177 | 9 | 10 | 187 | 94.7 (90.1–97.2) | 95.4 (91.2–97.7) |
| Chang,2023 | 616 | 143 | 55 | 663 | 91.8 (89.4–93.7) | 82.3 (79.4–84.8) |
| Chu,2019 | 60 | 1 | 0 | 64 | 100 (92.6–100) | 98.5 (90.6–99.8) |
| Javed,2022 | 12 | 1 | 2 | 13 | 85.7 (55.6–97.1) | 92.9 (63.7–99.3) |
| Mukherjee,2022 | 41 | 3 | 2 | 53 | 95.3 (82.9–99.0) | 94.6 (84.2–98.5) |
| Pooled (random‑effects) |  |  |  |  | 92.9 (91.0–94.9) | 92.7 (85.1–100)† |
| Heterogeneity I² (%) |  |  |  |  | 10.6 | 94.7 |
| Notes: TP = true positive, FP = false positive, FN = false negative, TN = true negative. A continuity correction (adding 0.5 to each cell) was applied for studies with zero cells. Pooled estimates wereobtained using a univariate random‑effects model (restricted maximum likelihood, REML).The upper bound of the 95% CI for pooled specificity was truncated at 100%. | | | | | | |
|  |  |  |  |  |  |  |
|  |  |  |  |  |  |  |
|  |  |  |  |  |  |  |
