# Supplementary material for: Deep learning models for pancreatic cancer detection on CT: a meta-analysis
Source: Front Med (Lausanne). 2026 Sep 14;13:1826557. doi: 10.3389/fmed.2026.1826557 (PMC13616657; doi:10.3389/fmed.2026.1826557)
Supplement: Supplementary file 5 [file Table_5.DOCX]

| Supplementary Table 4.Leave‑one‑out sensitivity analysis: changes in pooled sensitivity and specificity after removing each study. | | |
| --- | --- | --- |
|  |  |  |
| Study removed | Sensitivity | Specificity |
| Korfiatis2023 | 0.0034 | 0.0007 |
| Liu2020 | 0.0032 | –0.0045 |
| Chen2023 | 0.0023 | 0.0021 |
| Cao2023 | –0.0027 | –0.0078 |
| QU2024 | 0.0042 | 0.0037 |
| Ma2020 | 0.0009 | –0.0035 |
| Si2021 | 0.0035 | 0.0066 |
| Park2023 | 0.0034 | 0.0051 |
| Ramaekers2024 | –0.0053 | –0.0056 |
| Viriyasaranon2023 | 0.0027 | –0.0009 |
| Mandal2024 | 0.0015 | 0.0032 |
| Liao2025 | –0.0041 | 0.006 |
| Thanya2025 | –0.0081 | –0.0015 |
| Degand2025 | 0.0035 | 0.0018 |
| Nadeem2025 | –0.0070 | –0.0066 |
| Notes: Sensitivity / Specificity = (pooled estimate after removing the study) – (pooled estimate from all 15 studies). All absolute changes were well below the pre‑specified influential threshold of 0.02,  indicating that no single study disproportionately influenced the summary estimates. | | |
|  |  |  |
|  |  |  |
|  |  |  |
|  |  |  |
