# Supplementary material for: Deep learning models for pancreatic cancer detection on CT: a meta-analysis
Source: Front Med (Lausanne). 2026 Sep 14;13:1826557. doi: 10.3389/fmed.2026.1826557 (PMC13616657; doi:10.3389/fmed.2026.1826557)
Supplement: Supplementary file 6 [file Table_6.DOCX]

| Supplementary Table 5. Meta‑regression and publication bias results | | | | |
| --- | --- | --- | --- | --- |
|  |  |  |  |  |
| Analysis | Parameter | Estimate | 95% CI | p-value |
| Meta‑regression (logit sensitivity) | Design (multicenter vs. single‑center) | –0.587 | –1.085 to –0.088 | 0.021 |
|  | Model type (per increment: pure → hybrid → task‑enhanced) | 0.242 | –0.159 to 0.642 | 0.237 |
| Publication bias | Egger’s test (t) | 0.59 | – | 0.567 |
|  | Begg’s test (z) | 1.93 | – | 0.054 |
| Notes: Meta‑regression was performed using a random‑effects model (REML) on logit sensitivity.  Study design: 0 = single‑center, 1 = multicenter. Model type: 1 = pure (CNN/Transformer), 2 = hybrid, 3 = task‑enhanced. Publication bias tests were based on the diagnostic odds ratio (DOR).  Deeks’ funnel plot asymmetry test (p = 0.49) is reported in the main manuscript (Section 3.3). | | | | |
|  |  |  |  |  |
|  |  |  |  |  |
|  |  |  |  |  |
|  |  |  |  |  |
