# Supplementary material for: Deep learning models for pancreatic cancer detection on CT: a meta-analysis
Source: Front Med (Lausanne). 2026 Sep 14;13:1826557. doi: 10.3389/fmed.2026.1826557 (PMC13616657; doi:10.3389/fmed.2026.1826557)
Supplement: Supplementary file 7 [file Table_7.DOCX]

| Supplementary Table 6.Subgroup analysis of diagnostic performance by METRICS quality category | | | | | |
| --- | --- | --- | --- | --- | --- |
| Quality category | N | Pooled sensitivity | Pooled specificity | Difference (Good – Moderate) [95% CI] | p‑value |
| Good | 8 | 0.9 | 0.932 | Sensitivity: −0.0043 [−0.0582, 0.0529] | 0.89 |
| Moderate | 6 | 0.908 | 0.946 | Specificity: −0.0242 [−0.114, 0.0364] | 0.56 |
| Note: Bootstrap 5,000 resamples. The single low‑quality study was excluded. | | | | | |
